# Evaluation of Sleep Habits and Disturbances Among US Adults, 2017-2020

**DOI:** 10.1001/jamanetworkopen.2022.40788

**Published:** 2022-11-08

**Authors:** Hongkun Di, Yanjun Guo, Iyas Daghlas, Liang Wang, Gang Liu, An Pan, Liegang Liu, Zhilei Shan

**Affiliations:** 1 Department of Nutrition and Food Hygiene, Hubei Key Laboratory of Food Nutrition and Safety, School of Public Health, Tongji Medical College, Huazhong University of Science and Technology, Wuhan, China; 2 Ministry of Education Key Laboratory of Environment and Health, School of Public Health, Tongji Medical College, Huazhong University of Science and Technology, Wuhan, China; 3 Department of Occupational and Environmental Health, School of Public Health, Tongji Medical College, Huazhong University of Science and Technology, Wuhan, Hubei, China; 4 Department of Neurology, School of Medicine, University of California, San Francisco; 5 Department of Public Health, Robbins College of Health and Human Sciences, Baylor University, Waco, Texas; 6 Department of Epidemiology and Biostatistics, School of Public Health, Tongji Medical College, Huazhong University of Science and Technology, Wuhan, China

## Abstract

**Importance:**

Unhealthy sleep behaviors and sleep disturbances are associated with higher risk of multiple diseases and mortality. The current profiles of sleep habits and disturbances, particularly the differences between workdays and free days, are unknown in the contemporary US.

**Objective:**

To comprehensively evaluate sleep habits on workdays and free days and the prevalence of sleep disturbances among US adults.

**Design, Setting, and Participants:**

This study is a cross-sectional analysis of US nationally representative data from the National Health and Nutrition Examination Survey (2017-2020) among adults aged 20 years or older. Data analysis was performed from February to May 2022.

**Main Outcomes and Measures:**

The main outcomes were means and/or distributions of sleep habits, including sleep duration and sleep-wake timing on workdays and free days, sleep debt (ie, the difference between sleep duration on free days and mean weekly sleep duration), and social jet lag (ie, the difference between the midpoint between sleep and wake time on workdays and free days). Prevalence of trouble sleeping (ie, participants told a doctor or other health professional that they have trouble sleeping) and daytime sleepiness (ie, self-reported feeling of being overly sleepy during the day ≥5 times per month) were also determined.

**Results:**

A total of 9004 individuals (mean [SE] age, 48.3 [0.53] years; 4635 women [51.9%]; 3158 non-Hispanic White [62.8%]) were included in the current study. The mean sleep duration was 7.59 hours (95% CI, 7.54 to 7.64 hours) on workdays and 8.24 hours (95% CI, 8.17 to 8.31 hours) on free days (difference, 0.65 hour). The mean sleep and wake times were at 11:02 pm (95% CI, 10:57 pm to 11:17 pm) and 6:41 am (95% CI, 6:36 am to 6:45 am), respectively, on workdays and 11:25 pm (95% CI, 11:21 pm to 11:35 pm) and 7:41 am (95% CI, 7:37 am to 7:46 am), respectively, on free days (differences, 0.23 hour for sleep time and 1.00 hour for wake time). On workdays, 23.1% (95% CI, 21.3% to 24.9%) of adults slept less than 7 hours and 25.4% (95% CI, 24.1% to 26.6%) went to sleep at midnight or later; the corresponding percentages changed to 12.9% (95% CI, 11.6% to 14.1%) and 40.9% (95% CI, 38.4% to 43.5%), respectively, on free days. Furthermore, the mean sleep debt was 0.73 hours (95% CI, 0.68 to 0.77 hours), and mean social jet lag was 1.10 hours (95% CI, 1.05 to 1.15 hours); 30.5% (95% CI, 26.8% to 33.3%) of adults experienced 1 hour or more of sleep debt, and 46.5% (95% CI, 42.6% to 50.3%) experienced 1 hour or more of social jet lag. The prevalence of trouble sleeping was 29.8% (95% CI, 28.2% to 31.5%), and that of daytime sleepiness was 27.2% (95% CI, 25.0% to 29.5%).

**Conclusions and Relevance:**

In 2017 to 2020, US adults showed variability in sleep habits between workdays and free days, with longer sleep duration and later sleep-wake phases on free days, and high percentages of US adults experienced long-term sleep deprivation, chronic social jet lag, and frequent sleep disturbances. These findings provide evidence to further investigate potential approaches to optimize overall US sleep health.

## Key Points

**Question:**

What were the profiles of sleep habits and the prevalence of sleep disturbances among US adults in 2017 to 2020?

**Findings:**

In this nationally representative cross-sectional analysis with 9004 adults, differences in sleep patterns between workdays and free days were observed (0.65 hour for sleep duration, 0.23 hour for sleep time, and 1.00 hour for wake time). With regard to sleep disturbances, 30.5% of adults experienced 1 hour or more of sleep debt, 46.5% experienced 1 hour or more of social jet lag, 29.8% had trouble sleeping, and 27.2% experienced daytime sleepiness.

**Meaning:**

These findings elaborate on sleep habits and disturbances among US adults and provide evidence to further investigate potential approaches to optimize overall US sleep health.

## Teaser

This cross-sectional study evaluates sleep habits on workdays and free days and the prevalence of sleep disturbances among US adults.

## Introduction

Suboptimal sleep is associated with poor cognitive functioning, mental health, and cardiometabolic health.^1,2,3,4,5,6^ As a result of modern lifestyles, sleep habits of adults vary across workdays and free days.^7^ People commonly extend sleep duration on free days to compensate for sleep debt accumulated over the workweek or show irregular, weekly changes in sleep-wake timing, resulting in chronic sleep loss and circadian disruption.^8,9^ Consequently, it is crucial to comprehensively assess the current sleep habits and circadian characteristics among US adults, which could help address the sleep problems and their related health outcomes.

Several studies^10,11^ have reported the sleep duration among US adults only on workdays and the prevalence of sleep disturbances up to 2014. However, to our knowledge, no studies have explicitly investigated the sleep-wake timing and regularity of sleep habits across workdays and free days, all of which are essential components of healthy sleep. Therefore, by using nationally representative data from the National Health and Nutrition Examination Survey (NHANES) from 2017 to 2020, we evaluated whether the sleep duration and sleep-wake timing among US adults differed between workdays and free days and the extent to which they experienced sleep debt (ie, the difference between sleep duration on free days and mean weekly sleep duration), social jet lag (ie, the difference between the midpoint between sleep and wake time on workdays and free days), and sleep disturbances.

## Methods

### Study Population

NHANES is an ongoing, biennial, nationally representative series of surveys designed to monitor the health and nutritional status of the noninstitutionalized civilian US population^12^ that uses a complex, multistage, probability sampling design, with oversampling of different subpopulations to improve estimate accuracy. Because of the COVID-19 pandemic, field operations were suspended in March 2020, and data collected from 2019 to March 2020 were combined with data from the 2017 to 2018 cycle, forming a nationally representative sample of NHANES 2017 to March 2020 prepandemic data.^12^ Sample weights are provided to account for the complex survey design, including oversampling, survey nonresponse, and poststratification. The survey protocols were approved by the Centers for Disease Control and Prevention National Center for Health Statistics ethics review board, and additional details are available elsewhere.^13^ The study sample comprised adult subjects aged 20 years and older who responded to questions regarding sleep health contained in the interview components of 2017 to 2020 NHANES. All participants provided written informed consent at the time of the survey. Because we used publicly available data sets from NHANES, institutional review board approval and informed consent were not required for the current analysis, in accordance with 45 CFR §46. This cross-sectional study followed the Strengthening the Reporting of Observational Studies in Epidemiology (STROBE) reporting guideline.^14^

### Assessment of Sleep Habits and Disturbances

The Munich ChronoType Questionnaire was used to investigate the sleep-wake timing on workdays and free days (or weekdays and weekends for nonemployed individuals, including those who were retired, unemployed, and students).^15^ Sleep duration (hours) on workdays and free days was derived from reported sleep-wake timing, and absolute and relative sleep debt were calculated as the absolute and actual difference between sleep duration on free days and the mean weekly sleep duration, respectively.^16^ Midsleep time on workdays and free days was calculated as the midpoint between sleep and wake time, and the absolute and relative social jet lag were calculated as the absolute and actual difference between midsleep time on workdays and midsleep time on free days, respectively.^8,17^ The basic questionnaire items or computations of these sleep traits are described in eTable 1 in the Supplement. Sleep duration (<7 hours [short sleep], 7-9 hours, and ≥9 hours [long sleep]), sleep time (before 10:00 pm [early bedtime], 10:00 pm to midnight, midnight or later [late bedtime]), and wake time (before 6:00 am [early wake time], 6:00 am to 8:00 am, 8:00 am or later [late wake time]) were further defined. Sleep debt and social jet lag were both expressed as absolute differences and were further dichotomized as low (<2 hours) or high (≥2 hours) for sleep debt and as mild (<2 hours) or heavy (≥2 hours) for social jet lag, as done in prior studies.^8,17,18,19^

The sleep disturbances questions by in-home interviews fall into 3 general categories: breathing problem during sleep, sleep difficulty, and daytime sleepiness. Given the investigation method that the interviewers directly asked the participants themselves, this study only included trouble sleeping and daytime sleepiness as sleep disturbances for the data reliability and clinical relevance. Trouble sleeping was defined as the participants told a doctor or other health professional that they have trouble sleeping. Daytime sleepiness was defined as self-reported feeling of being overly sleepy during the day 5 or more times per month. The questions, values, and categories are presented in eTable 1 in the Supplement.

### Assessment of Sociodemographic and Employment Variables

A standardized questionnaire was used to collect information on sex, age, race, ethnicity, education level, and family income. Information on race and ethnicity was self-reported by NHANES participants according to categories provided by the National Center for Health Statistics (Mexican American, non-Hispanic Black, non-Hispanic White, other Hispanic, or other). Mexican American and other Hispanic groups were combined to create the Hispanic group. Other includes any other race or ethnicity other than non-Hispanic White, non-Hispanic Black, or Hispanic. This information was collected to evaluate sleep habits and disturbances by race and ethnicity. Age was stratified as 20 to 39, 40 to 59, 60 to 74, and 75 or more years old. Education was grouped into a categorical variable, with categories as follows: less than high school, high school, and greater than high school. Family income levels were classified into 3 categories: ratio of family income to poverty level less than 1.30, 1.30 to 3.49, and 3.50 or higher.^20^ The Occupation Questionnaire was used to collect and create the following employment information: work status (nonemployed [including the unemployed, retirees, students, and those not actively looking for work], part time [1-34 hours per week], and full time [≥35 hours per week]), and work schedule (traditional 9 am to 5 pm day, regular shift work [evening or nights or early mornings], rotating shift work [early mornings, days, and nights], and did not work).^21^

### Statistical Analysis

Sample weights, clustering, and stratification were incorporated in all analyses because of the complex sampling design of the NHANES, as required to analyze the NHANES data.^22^ Data are reported as weighted and nationally representative estimates of means and frequencies, unless otherwise stated.

First, estimates of the weighted means and/or distributions of sleep duration, sleep-wake timing, sleep debt, and social jet lag, and the weighted prevalence of sleep disturbances were examined in the total population and by major population subgroups, including age, sex, race and ethnicity, education, income level, work status, and work schedule. All 95% CIs were estimated using SEs obtained by Taylor series linearization.^23,24^ Sample means were compared using analysis of variance, and sample prevalence or proportions were compared using Rao-Scott χ^2^ tests.^24^ In sensitivity analysis, sleep debt was redefined as the absolute difference in sleep duration between work and free days, aiming to illustrate the distribution of sleep debt accumulating during the workweek by sleeping in on free days. Second, estimated odds ratios (ORs) for the association of covariates with sleep characteristics mentioned above were identified using multivariable-adjusted logistic regression models. The testing global null hypothesis was set as β = 0. T trends across subgroups of age, education, and family income level were calculated by including these stratification factors as continuous variables in the logistic regression models.

Data analysis was performed from February to May 2022. All data analyses were conducted using survey procedures in SAS statistical software version 9.4 (SAS Institute). All statistical tests were performed with a significance level of 2-sided *P* < .05.

## Results

### Participant Characteristics

After excluding 210 participants (2.27%) with missing data on sleep-wake timing and 18 (0.19%) with missing data on sleep disturbances, a total of 9004 individuals (mean [SE] age, 48.3 [0.53] years; 4635 women [51.9%]; 3158 non-Hispanic White [62.8%]) were analyzed. Unweighted sample sizes overall by sociodemographic and employment characteristics are presented in Table 1. The basic characteristics of participants included and excluded in the analyses are presented in eTable 2 in the Supplement. Compared with individuals excluded, those included were of similar age and sex, but were more likely to be non-Hispanic White, full-time workers with a 9 am to 5 pm schedule, and to have higher education and family income levels.

**Table 1.** Participant Characteristics for Sleep Habits and Sleep Disturbances Among US Adults Aged 20 Years or Older by Sociodemographic Characteristics, National Health and Nutrition Examination Survey 2017-2020

Characteristics	**Participants, No. (Weighted %) (N = 9004)** ^a^
Age, y	
20-39	2753 (36.1)
40-59	2929 (34.0)
60-74	2279 (21.5)
≥75	1043 (8.34)
Sex	
Women	4635 (51.9)
Men	4369 (48.1)
Race and ethnicity	
Hispanic^b^	1961 (16.0)
Non-Hispanic Black	2355 (11.2)
Non-Hispanic White	3158 (62.8)
Other^c^	1530 (10.0)
Educational attainment	
Less than high school graduate	1704 (10.9)
High school graduate or general equivalency diploma	2146 (26.7)
Some college or above	5141 (62.4)
Ratio of family income level to poverty level	
<1.30	2135 (16.4)
1.30-3.49	3021 (30.5)
≥3.50	1339 (40.7)
Work status	
Nonemployed	3996 (37.2)
Part time (1-34 h/wk)	1292 (15.6)
Full time (≥35 h/wk)	3706 (47.3)
Work schedule	
Traditional 9 am to 5 pm day	1972 (26.7)
Shift work	
Regular	1383 (14.5)
Rotating	1774 (23.3)
Did not work	3875 (35.5)

^a^ Numbers of participants are unweighted. All percentage estimates are weighted.

^b^ Includes Mexican Americans.

^c^ Other includes any other race or ethnicity other than non-Hispanic White, non-Hispanic Black, or Hispanic.

### Sleep Duration and Sleep-Wake Timing

**Figure 1.**
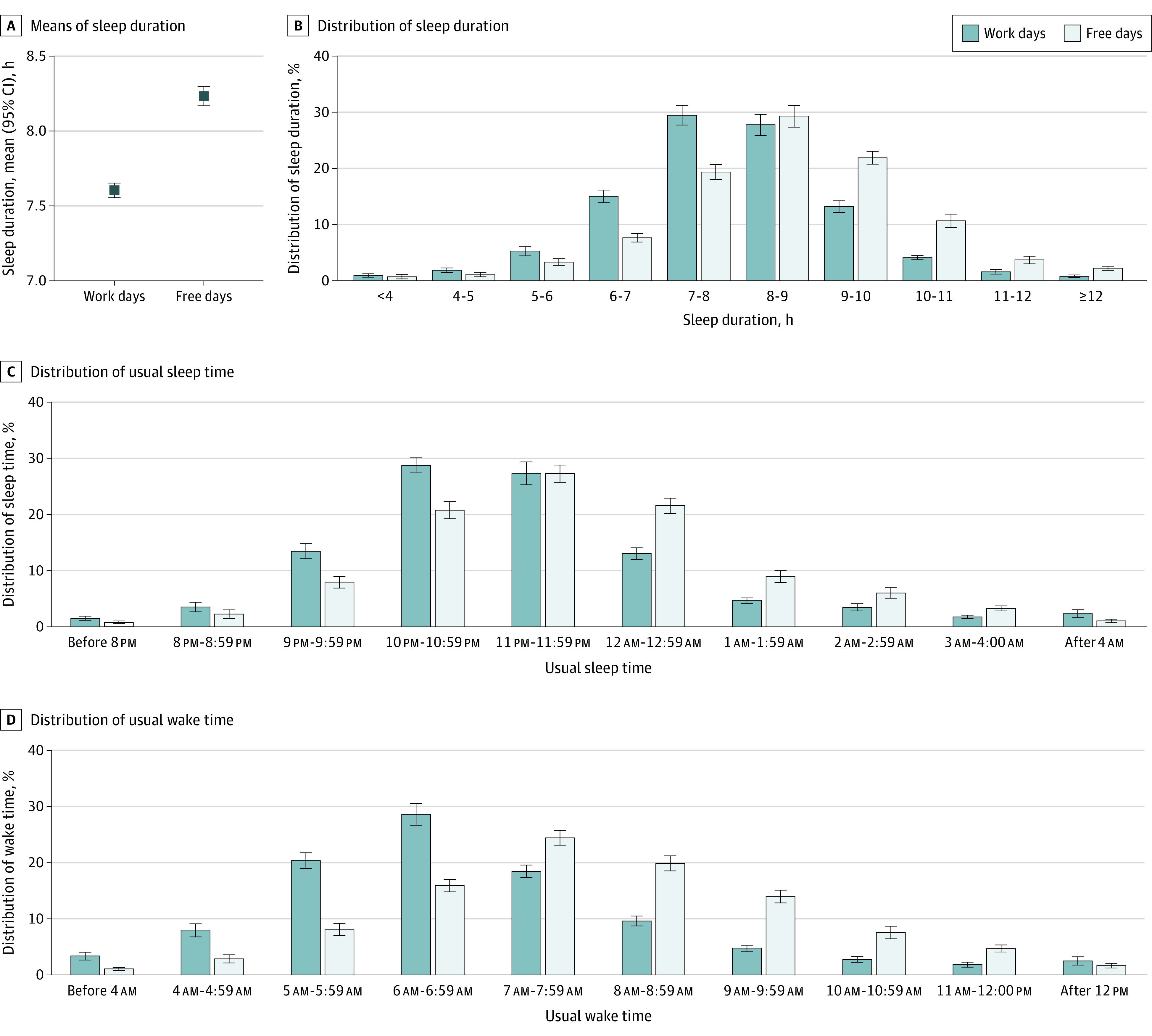
Distributions of Sleep Duration and Sleep-Wake Timing

In 2017 to 2020, the mean sleep duration was significantly longer on free days than on workdays (8.24 hours [95% CI, 8.17-8.31 hours] vs 7.59 hours [95% CI, 7.54-7.64 hours]; difference, 0.65 hour [95% CI, 0.63-0.67 hour]; *P* < .001) (Figure 1A and eTable 3 in the Supplement). On workdays, 23.1% (95% CI, 21.3%-24.9%) of adults had short sleep duration (<7 hours) and 19.7% (95% CI, 18.5%-21.0%) had long sleep duration (≥9 hours). On free days, 12.9% (95% CI, 11.6%-14.1%) of adults had short sleep duration and 38.5% (95% CI, 36.7%-40.3%) had long sleep duration (Figure 1B).

Sleep-wake timing also differed markedly across workdays and free days. On workdays, the mean sleep time was 11:02 pm (95% CI, 10:57 pm to 11:17 pm) and the mean wake time was 6:41 am (95% CI, 6:36 am to 6:45 am), whereas on free days, the mean sleep time was 11:25 pm (95% CI, 11:21 pm to 11:35 pm) and the mean wake time was 7:41 am (95% CI, 7:37 am to 7:46 am) (*P* < .001) (eTable 4 and eTable 5 in the Supplement). On workdays, 18.5% (95% CI, 16.8% to 20.2%) of adults fell asleep before 10:00 pm and 25.4% (95% CI, 24.1% to 26.6%) did so at midnight or later; 31.6% (95% CI, 29.8% to 33.5%) of adults woke up before 6:00 am and 21.4% (95% CI, 20.1% to 22.6%) woke up at 8:00 am or later (Figures 1C and 1D). On free days, 11.0% (95% CI, 9.70% to 12.3%) of adults fell asleep before 10:00 pm and 40.9% (95% CI, 38.4% to 43.5%) did so at midnight or later; 12.0% (95% CI, 10.7% to 13.4%) of adults woke up before 6:00 am, and 47.7% (95% CI, 45.6% to 49.7%) woke up at 8:00 am or later (Figures 1C and 1D). Midsleep times also showed a similar frequency distribution: the largest binned group shifted to later hours on free days (eFigure 1 in the Supplement).

### Sleep Debt, Social Jet Lag, and Sleep Disturbances

**Figure 2.**
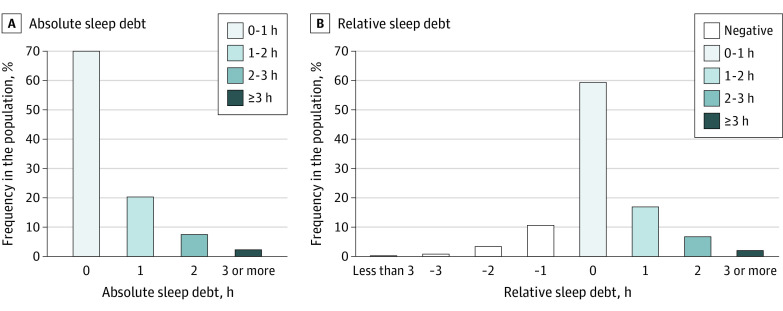
Distributions of Absolute and Relative Sleep Debt Sleep debt is defined as the difference between the average weekday and free-day sleep duration. The lowest bins of sleep debt represent less than 1 hour and less than 3 hours, the highest bins of sleep debt represent 3 hours or more, and intermediary bins include the lower and exclude the upper limit.

**Figure 3.**
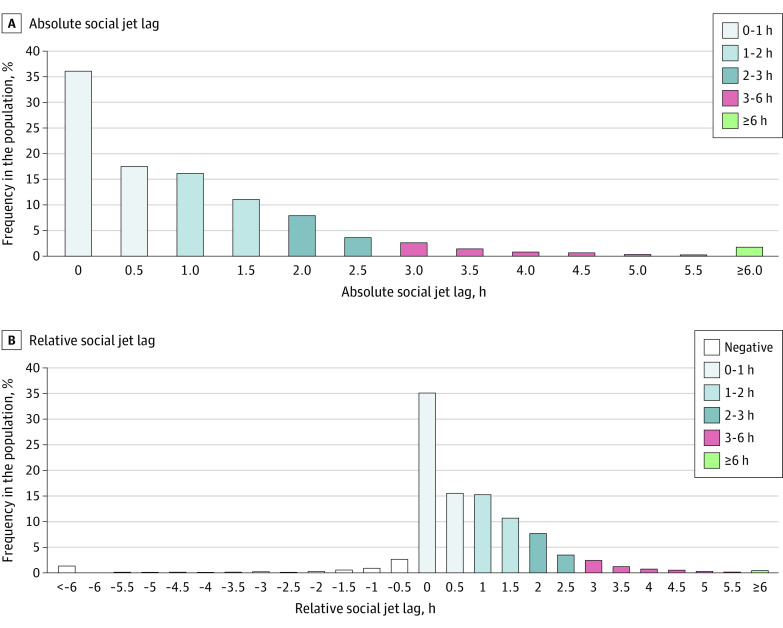
Distributions of Absolute and Relative Social Jet Lag The distribution is based on half-hour bins, except for the categories at both ends. The lowest bins of social jet lag represent less than 1 hour and less than 6 hours, the highest bins of social jet lag represent 6 hours or more, and intermediary bins include the lower and exclude the upper limit.

The mean sleep debt was 0.73 hour (95% CI, 0.68-0.77 hour); 30.5% (95% CI, 26.8%-33.3%) of adults accumulated at least 1 hour of sleep debt per week, and 9.75% (95% CI, 8.65%-10.8%) accumulated at least 2 hours per week (Figure 2 and eTable 6 in the Supplement); 15.0% (95% CI, 13.0%-16.9%) had negative relative sleep debt (Figure 2). Similar distributions were observed when sleep debt was expressed as the absolute difference in sleep duration between work and free days, with the least sleep debt among older adults (eTable 7 and eFigure 2 in the Supplement). The mean social jet lag was 1.10 hours (95% CI, 1.05-1.15 hours), 46.5% (95% CI, 42.6%-50.3%) of adults had at least 1 hour of social jet lag per week, and 19.3% (95% CI, 17.7%-20.9%) had at least 2 hours jet lag per week (Figure 3 and eTable 8 in the Supplement); 6.54% (95% CI, 4.09%-9.04%) had negative relative social jet lag (Figure 3). The estimated prevalence of trouble sleeping was 29.8% (95% CI, 28.2%-31.5%), and that of daytime sleepiness was 27.2% (95% CI, 25.0%-29.5%) (Table 2).

**Table 2.** Estimated Prevalence and Adjusted Relative Odds of Sleep Disturbances Among US Adults Aged 20 Years or Older, National Health and Nutrition Examination Survey 2017-2020^a^

Characteristics	Weighted % (95% CI)				Adjusted OR (95% CI)^b^			
	Trouble sleeping	*P* value^c^	Daytime sleepiness^d^	*P* value^c^	Trouble sleeping	*P* value for trend^e^	Daytime sleepiness^d^	*P* value for trend^e^
Overall	29.8 (28.2-31.5)		27.2 (25.0-29.5)		NA		NA	
Age, y								
20-39	23.2 (21.2-25.1)	<.001	31.7 (28.8-34.6)	<.001	1 [Reference]	.04	1 [Reference]	<.001
40-59	32.7 (29.8-35.6)		24.5 (21.4-27.6)		1.62 (1.37-1.92)		0.71 (0.61-0.82)	
60-74	37.3 (33.7-40.8)		24.3 (21.2-27.4)		1.44 (1.21-1.71)		0.61 (0.51-0.74)	
≥75	28.0 (24.2-31.8)		26.8 (23.8-29.8)		0.74 (0.62-0.89)		0.60 (0.47-0.78)	
Sex								
Female	33.0 (30.5-35.6)	<.001	30.1 (27.9-32.3)	<.001	1 [Reference]	NA	1 [Reference]	NA
Male	26.4 (24.4-28.5)		24.2 (20.9-27.4)		0.80 (0.68-0.93)		0.75 (0.62-0.91)	
Race and ethnicity								
Hispanic^f^	23.3 (20.2-26.4)	<.001	23.2 (19.6-26.8)	<.001	0.64 (0.50-0.81)	NA	0.60 (0.48-0.76)	NA
Non-Hispanic Black	24.7 (22.1-27.2)		23.7 (21.3-26.2)		0.64 (0.53-0.76)		0.61 (0.50-0.76)	
Non-Hispanic White	33.2 (30.7-35.7)		29.9 (26.9-32.9)		1 [Reference]		1 [Reference]	
Other^g^	24.9 (20.8-28.9)		21.2 (17.2-25.2)		0.67 (0.52-0.86)		0.59 (0.43-0.81)	
Educational attainment								
Less than high school	26.4 (22.3-30.6)	.24	24.8 (22.1-27.5)	.06	1 [Reference]	.05	1 [Reference]	.55
High school	30.7 (27.5-33.8)		29.5 (25.7-33.3)		1.22 (0.95-1.55)		1.17 (0.96-1.42)	
More than high school	30.1 (28.1-32.1)		26.7 (24.2-29.2)		1.28 (1.03-1.58)		1.13 (0.94-1.35)	
Ratio of family income level to poverty level								
<1.30	31.4 (27.3-35.6)	.52	33.1 (28.9-37.3)	<.001	1 [Reference]	.03	1 [Reference]	<.001
1.30-3.49	30.6 (27.6-33.6)		31.0 (27.8-34.3)		0.95 (0.77-1.16)		0.92 (0.80-1.05)	
≥3.50	29.3 (26.7-31.8)		23.4 (20.6-26.1)		0.83 (0.68-1.01)		0.61 (0.49-0.76)	
Work status								
Nonemployed	38.0 (35.4-40.7)	<.001	29.4 (26.6-32.1)	.13	0.97 (0.50-1.89)	NA	1.71 (1.03-2.85)	NA
Part time (1-34 h/wk)	26.7 (22.2-31.2)		26.5 (21.4-31.6)		1 [Reference]		1 [Reference]	
Full time (≥35 h/wk)	24.5 (22.0-26.9)		25.8 (23.0-28.6)		0.91 (0.68-1.22)		1.10 (0.82-1.48)	
Work schedule								
Traditional 9 am to 5 pm day	25.0 (21.2-28.7)	<.001	23.0 (19.2-26.9)	.02	1 [Reference]	NA	1 [Reference]	NA
Regular shift work	23.4 (19.2-27.6)		28.1 (24.1-32.1)		1.01 (0.71-1.43)		1.31 (0.99-1.75)	
Rotating shift work	26.2 (22.5-29.8)		28.7 (24.2-33.2)		1.06 (0.78-1.44)		1.32 (0.96-1.83)	
Did not work	38.6 (36.0-41.1)		29.1 (26.6-31.6)		1.90 (1.09-3.33)		0.90 (0.53-1.54)	

Abbreviation: NA, not applicable.

^a^ Weighted estimates and 95% CIs were estimated for each stratum. All estimates were weighted to be nationally representative.

^b^ Akaike information criterion was 277 369 550 for trouble sleeping and 269 090 554 for daytime sleepiness.

^c^ *P* values are shown for overall differences across stratums.

^d^ Daytime sleepiness was defined as the self-reported feeling of being overly sleepy often (5-15 times a month) or always (16-30 times a month) during the day.

^e^ Calculated using logistic regression models that included the median value of each category of age or family income level as a continuous variable, and included the education level as a continuous variable.

^f^ Includes Mexican Americans.

^g^ Includes any race or ethnicity other than non-Hispanic White, non-Hispanic Black, or Hispanic.

### Subgroup and Multivariable Analysis

In univariable analyses, consistent differences in sleep duration or of the proportion of normal bedtime to wake time between workdays and free days were observed in most subgroups, except for participants aged 75 years and older (eTable 3, eTable 4, and eTable 5 in the Supplement). The percentages of adults with high sleep debt (≥2 hours) and heavy social jet lag (≥2 hours) were both higher among younger adults, non-Hispanic Black individuals, full-time workers, and regular shift workers (eTable 6 and eTable 8 in the Supplement). Trouble sleeping was more prevalent among older adults, women, non-Hispanic White individuals, and nonemployed people, whereas daytime sleepiness was more prevalent among younger adults, women, non-Hispanic White individuals, nonemployed people, and those with lower income level (Table 2).

After multivariable adjustment, age, sex, race, ethnicity, work status, and work schedule were associated with short sleep duration on workdays; age, sex, race, ethnicity, education, and work schedule were associated with long sleep duration on free days; and age, race, ethnicity, family income level and work schedule were associated with late bedtime (eTable 9 and eTable 10 in the Supplement). Age, race, ethnicity, family income level, work status, and schedule were associated with both high sleep debt and heavy social jet lag (eTable 11 in the Supplement). Age, sex, race, ethnicity, educational level, and work schedule were associated with trouble sleeping, whereas age, sex, race, ethnicity, family income level and work status were associated with daytime sleepiness (Table 2).

## Discussion

This cross-sectional study found that in 2017 to 2020, US adults had distinct sleep habits on workdays and free days, with a 0.65-hour longer sleep duration, a 0.23-hour later sleep time, and a 1.00-hour later wake time on free days; 30.5% and 46.5% of adults experienced at least 1 hour of sleep debt and social jet lag, respectively. The prevalence of trouble sleeping and daytime sleepiness were 29.8% and 27.2%, respectively.

To our knowledge, this is the first study to evaluate the sleep duration among nationally representative US adults on workdays and free days separately. In our study, the estimated sleep duration was 7.59 hours and the proportion of adults with short sleep (<7 hours) and long sleep (≥9 hours) were 23.1% and 19.7%, respectively, on workdays. These findings were not consistent with previous studies^10,11,25^ showing a sleep duration of 7.18 hours in 2012 among US adults aged 18 years and older and 7.00 hours in 2014 among adults aged 16 years and older. Consistently, the prevalence of adults with sleep shorter than 7 hours in this study was lower than prior estimates from the National Health Interview Survey (29.2% in 2012) and from the Behavioral Risk Factors Surveillance System (35.0% in 2014 and 13.0% in 2018).^10,25,26^ The differences could be due to differences in the questionnaires, since participants in NHANES before 2015, or National Health Interview Survey and Behavioral Risk Factors Surveillance System, were required to self-report round numbers, whereas estimates were rounded to the nearest half-hour in NHANES 2017 to 2020. Moreover, we first estimated that the sleep debt among US adults was 0.73 hours, 30.5% of adults experienced at least 1 hour of sleep debt, and 9.75% experienced at least 2 hours of sleep debt. Given that emerging evidence suggests that, in addition to irregular sleep duration, sleep debt could be associated with obesity, diabetes, cardiovascular health, and mood disorders,^5,27,28,29^ more evidence is needed on the trend in sleep debt over time and the benefits of reducing its risk, even in those without substantive sleep deprivation.

We provided an overall profile of sleep-wake patterns and the differences between workdays and free days among US adults. The sleep-wake timing was much later on free days (11:25 pm to 7:41 am) than on workdays (11:02 pm to 6:41 am); the proportion of late bedtime (midnight or later) increased from 25.4% to 40.9% (15.5% increase), and the proportion of late wake time (8:00 am or later) increased from 21.4% to 47.7% (26.3% increase). This was in line with a previous study^30^ that identified within-week patterns of bedtime among US adults in 2003 to 2006, showing varying degrees of delay in the midpoint of bedtime on weekend nights, compared with weekday nights. In addition, our results showed a higher percentage of late bedtime among US adults in 2017 to 2020 than estimates in a cohort study^31^ from 21 other high-income countries reporting that 18.5% of participants aged 35 to 70 years slept at midnight or later during 2003 to 2009. Additionally, we first estimated that the social jet lag among US adults was 1.10 hours, and 46.5% of adults were estimated to have experienced at least 1 hour of social jet lag, with 19.3% having experienced at least 2 hours. A previous large-scale epidemiological study^8^ of more than 65 000 European participants showed that 33% of the population had 2 hours or more of social jet lag, and 69% reported at least 1 hour from 2002 to 2010. A growing body of evidence suggests that maintaining optimal sleep-wake timing and improving the correspondence between biological and social clocks may be an important consideration in the management of obesity, diabetes, and cardiovascular disease.^8,9,32,33^ More research is needed to determine long-term trends and further elucidate the importance of instability in sleep timing for public health.

Sleep disturbances remain highly prevalent among US adults in our study: 29.8% of adults had trouble sleeping and 27.2% experienced daytime sleepiness (via the NHANES Sleepiness questionnaire). The prevalence of trouble sleeping and daytime sleepiness was both higher than prior estimates. On the basis of data from adults aged 18 years and older from the National Health Interview Survey from 2002 to 2012, the prevalence of trouble sleeping increased from 17.5% to 19.2%, and excessive daytime sleepiness increased from 9.80% to 12.7%.^34^ Cross-sectional data of NHANES showed that the prevalence of trouble sleeping in 2005 to 2018 was 27.74%, and the prevalence of excessive daytime sleepiness in 2005 to 2008 was 18.5%.^35,36^ These increases may have been influenced by single-item questionnaires in our study, which used a broader definition. Still, these results underscore that sleep disturbances remain a major challenge for promotion of healthy sleep in the US.

The small differences in sleep duration and sleep-wake timing between workdays and free days among elderly individuals (ie, those aged ≥75 years) could be because most older adults did not work. Consistent with previous study, substantially higher prevalence of short sleep duration on workdays, long sleep duration on free days, late bedtime, high sleep debt, or heavy social jet lag was observed among non-Hispanic Black individuals, full-time workers, and regular shift workers.^37^ Working the night shift or long hours were probably the most relevant variables associated with changes in normal circadian rhythm, thus depriving individuals of a normal sleep cycle.^38^ The higher proportion of night shift workers among the non-Hispanic Black individuals could partly account for the association between race and ethnicity and sleep problems.^39^ Additional studies are warranted to further understand whether certain groups with poor sleep may be affected disproportionately.

### Strengths and Limitations

The strengths of this study include the use of a large, nationally representative survey and the separate assessments of sleep duration and sleep-wake timing on workdays and free days. This study also has several limitations. First, information on sleep data was self-reported rather than objectively measured and, therefore, was prone to misreporting and recall bias. Second, participants with missing data on sleep were excluded, which may affect the national representativeness of these findings. Third, in calculating mean weekly sleep duration, we assumed that most individuals followed a weekly structure of 5 weekdays or workdays and 2 weekends or free days. The part-time workers may not follow this structure, but they were the smallest subgroup of participants. Fourth, though social jet lag has been widely used as a measure of circadian misalignment, it could not fully accurately represent the degree of circadian disruption. Fifth, the question included in the NHANES sleepiness questionnaire was not validated. Future studies are needed with the standard questionnaires of sleep disturbances. Sixth, because of the unavailability of data, we failed to consider the impacts of seasonality of agricultural work and standard time clock changes on sleep habits, nor did we evaluate subjective sleep satisfaction.

## Conclusions

In this cross-sectional study, in 2017 to 2020, US adults showed variability in sleep habits across a week, with longer sleep duration and later sleep-wake phase on free days. A high percentage of US adults experienced long-term sleep deprivation, chronic social jet lag, and frequent sleep disturbances. These findings provide evidence to further investigate potential approaches to optimize the overall US sleep health.

## Notes

Supplement. **eTable 1.** Assessments of Sleep Characteristics, NHANES 2017-2020 **eTable 2.** Basic Characteristics of Participants Included and Excluded in the Survey **eTable 3. **Estimated Means and Distributions of Sleep Duration During Work Days and Free Days Among US Adults Aged 20 Years or Older, NHANES 2017-2020 **eTable 4.** Estimated Means and Distributions of Usual Sleep Time During Work Days and Free Days Among US Adults Aged 20 Years or Older, NHANES 2017-2020 **eTable 5.** Estimated Means and Distributions of Usual Wake Time During Work Days and Free Days Among US Adults Aged 20 Years or Older, NHANES 2017-2020 **eFigure 1.** Distributions of Sleep Debt (Absolute Difference in Sleep Duration Between Work and Free Days) **eTable 6.** Estimated Means and Distributions of Sleep Debt (Absolute Difference Between the Average Weekday and Free-Day Sleep Duration) Among US Adults Aged 20 Years or Older, NHANES 2017-2020 **eTable 7.** Estimated Means and Distributions of Sleep Debt (Absolute Difference in Sleep Duration Between Work and Free Days) Among US Adults Aged 20 Years or Older, NHANES 2017-2020 **eFigure 2.** Distributions of Mid-Sleep Time: Mid-Sleep on Work Days (MSW) and Free Days (MSF) With No Adjustments **eTable 8.** Estimated Means and Distributions of Social Jetlag (Sleep Midpoint Difference on Free-Days vs Work-Days) Among US Adults Aged 20 Years or Older, NHANES 2017-2020 **eTable 9.** Weighted Logistic Regression Models of Sleep Duration Among US Adults Aged 20 Years or Older, Adjusted for Sociodemographic and Employment Characteristics, NHANES 2017-2020 **eTable 10.** Weighted Logistic Regression Models of Sleep-Wake Timing Among US Adults Aged 20 Years or Older, Adjusted for Sociodemographic and Employment Characteristics, NHANES 2017-2020 **eTable 11.** Weighted Logistic Regression Models of Sleep Debt and Social Jetlag Among US Adults Aged 20 Years or Older Adjusted for Sociodemographic and Employment Characteristics, NHANES 2017-2020
eReferences
