# Supplementary material for: Evaluation of Sleep Habits and Disturbances Among US Adults, 2017-2020
Source: JAMA Netw Open. 2022 Nov 8;5(11):e2240788. doi: 10.1001/jamanetworkopen.2022.40788 (PMC9644264; doi:10.1001/jamanetworkopen.2022.40788)
Supplement: Supplement. — eTable 1. Assessments of Sleep Characteristics, NHANES 2017-2020 eTable 2. Basic Characteristics of Participants Included and Excluded in the Survey eTable 3. Estimated Means and Distributions of Sleep Duration During Work Days and Free Days Among US Adults Aged 20 Years or Older, NHANES 2017-2020 eTable 4. Estimated Means and Distributions of Usual Sleep Time During Work Days and Free Days Among US Adults Aged 20 Years or Older, NHANES 2017-2020 eTable 5. Estimated Means and Distributions of Usual Wake Time During Work Days and Free Days Among US Adults Aged 20 Years or Older, NHANES 2017-2020 eFigure 1. Distributions of Sleep Debt (Absolute Difference in Sleep Duration Between Work and Free Days) eTable 6. Estimated Means and Distributions of Sleep Debt (Absolute Difference Between the Average Weekday and Free-Day Sleep Duration) Among US Adults Aged 20 Years or Older, NHANES 2017-2020 eTable 7. Estimated Means and Distributions of Sleep Debt (Absolute Difference in Sleep Duration Between Work and Free Days) Among US Adults Aged 20 Years or Older, NHANES 2017-2020 eFigure 2. Distributions of Mid-Sleep Time: Mid-Sleep on Work Days (MSW) and Free Days (MSF) With No Adjustments eTable 8. Estimated Means and Distributions of Social Jetlag (Sleep Midpoint Difference on Free-Days vs Work-Days) Among US Adults Aged 20 Years or Older, NHANES 2017-2020 eTable 9. Weighted Logistic Regression Models of Sleep Duration Among US Adults Aged 20 Years or Older, Adjusted for Sociodemographic and Employment Characteristics, NHANES 2017-2020 eTable 10. Weighted Logistic Regression Models of Sleep-Wake Timing Among US Adults Aged 20 Years or Older, Adjusted for Sociodemographic and Employment Characteristics, NHANES 2017-2020 eTable 11. Weighted Logistic Regression Models of Sleep Debt and Social Jetlag Among US Adults Aged 20 Years or Older Adjusted for Sociodemographic and Employment Characteristics, NHANES 2017-2020 eReferences [file jamanetwopen-e2240788-s001.pdf]

Supplemental Online Content

Di H, Guo Y, Daghlas I, et al. Evaluation of sleep habits and disturbances among US adults, 2017-2020. *JAMA Netw Open*. 2022;5(11):e2240788. doi:10.1001/jamanetworkopen.2022.40788

eTable 1. Assessments of Sleep Characteristics, NHANES 2017-2020

eTable 2. Basic Characteristics of Participants Included and Excluded in the Survey

eTable 3. Estimated Means and Distributions of Sleep Duration During Work Days and Free Days Among US Adults Aged 20 Years or Older, NHANES 2017-2020

eTable 4. Estimated Means and Distributions of Usual Sleep Time During Work Days and Free Days Among US Adults Aged 20 Years or Older, NHANES 2017-2020

eTable 5. Estimated Means and Distributions of Usual Wake Time During Work Days and Free Days Among US Adults Aged 20 Years or Older, NHANES 2017-2020

eFigure 1. Distributions of Sleep Debt (Absolute Difference in Sleep Duration Between Work and Free Days)

eTable 6. Estimated Means and Distributions of Sleep Debt (Absolute Difference Between the Average Weekday and Free-Day Sleep Duration) Among US Adults Aged 20 Years or Older, NHANES 2017-2020

eTable 7. Estimated Means and Distributions of Sleep Debt (Absolute Difference in Sleep Duration Between Work and Free Days) Among US Adults Aged 20 Years or Older, NHANES 2017-2020

© 2022 Di H et al. *JAMA Network Open.*

eFigure 2. Distributions of Mid-Sleep Time: Mid-Sleep on Work Days (MSW) and Free Days (MSF) With No Adjustments

eTable 8. Estimated Means and Distributions of Social Jetlag (Sleep Midpoint Difference on Free-Days vs Work-Days) Among US Adults Aged 20 Years or Older, NHANES 2017-2020

eTable 9. Weighted Logistic Regression Models of Sleep Duration Among US Adults Aged 20 Years or Older, Adjusted for Sociodemographic and Employment Characteristics, NHANES 2017-2020

eTable 10. Weighted Logistic Regression Models of Sleep-Wake Timing Among US Adults Aged 20 Years or Older, Adjusted for Sociodemographic and Employment Characteristics, NHANES 2017-2020

eTable 11. Weighted Logistic Regression Models of Sleep Debt and Social Jetlag Among US Adults Aged 20 Years or Older Adjusted for Sociodemographic and Employment Characteristics, NHANES 2017-2020

eReferences

This supplemental material has been provided by the authors to give readers additional information about their work.

© 2022 Di H et al. *JAMA Network Open.*

eTable 1. Assessments of Sleep Characteristics, NHANES 2017-2020

Variables	Assessments (Questionnaire items/definitions)	Format	Weekdays or workdays		Weekends or work-free days	
			**Abbreviation**	**Computation**	**Abbreviation**	**Computation**
**Basic variables**						
Usual sleep time (sleep onset)	What time {do you/does SP} usually fall asleep on weekdays or workdays?<br>What time {do you/does SP} usually fall asleep on weekends or non-workdays?	hh:mm	SO_W	-	SO_F	-
Usual wake time (sleep end)	What time {do you/does SP} usually wake up on weekdays or workdays?<br>What time {do you/does SP} usually wake up on weekends or non-workdays?	hh:mm	SE_W	-	SE_F	-
Trouble sleeping	The response to {Have you/Has SP} ever told a doctor or other health professional that {you have/s/he has} trouble sleeping? was used to assess trouble sleeping.	-	-	-	-	-
Daytime sleepiness	Participants were asked "In the past month, how often did {you/SP} feel excessively or overly sleepy during the day?" with options of "Never, Rarely (1 time a month), Sometimes (2–4 times a month), Often (5–15 times a month) and Almost Always (16–30 times a month)"; responses were recoded dichotomously with ≥5 times/month being considered an indicator of daytime sleepiness.	-	-	-	-	-
**Computed variables**						

© 2022 Di H et al. *JAMA Network Open.*

Sleep duration	Number of hours usually sleep.	h	SD_W	SEw - SOw	SD_F	$SE_F - SO_F$
Mid-Sleep time	The midpoint between sleep time and wake time.	hh:mm	MSW	$SO_W + SD_W/2$	MSF	$SO_F + SD_F/2$

Computed variables combining workdays and work-free days

Variables	Assessments (Questionnaire items/definitions)	Format	Abbreviation	Computation
Average weekly sleep duration	A weighted average estimate of actual sleep duration between work and free days [(sleep duration on workdays*5 + sleep duration on free days*2) / 7], under the assumption that most individuals followed a weekly structure of 5 weekdays/workdays and 2 weekends/free days.	h	SD_{week}	$(SD_w * 5 + SD_F * 2)/7$
Absolute sleep debt	Absolute difference between sleep duration on free days and the average sleep duration.	h	-	$\|SD_F-SD_{week}\|$
Relative sleep debt	Actual difference between sleep duration on free days and the average sleep duration.	h	-	SD_F-SD_{week}
Absolute social jetlag	Absolute difference between MSW and MSF.	hh:mm	SJL	|MSF - MSW|
Relative social jetlag	Actual difference between MSF and MSW.	hh:mm	SJL_{rel}	MSF - MSW

© 2022 Di H et al. *JAMA Network Open.*

eTable 2. Basic Characteristics of Participants Included and Excluded in the Survey[a]

	Participants excluded (N = 228)		Participants included (N = 9004)		*P value*
	N	Mean (SD) or N(%)	N	Mean (SD) or N(%)	
**Age at survey (years)**	228	50.8 (1.33)	9004	48.3 (0.53)	.07
**Age (years)**	228		9004		.12
20-39		46 (28.7)		2753 (36.1)	
40-59		82 (42.4)		2929 (34.0)	
60-74		72 (18.9)		2279 (21.5)	
≥75		28 (9.95)		1043 (8.34)	
**Sex**	228		9004		.72
Female		118 (53.3)		4635 (51.9)	
Male		110 (46.7)		4369 (48.1)	
**Race/ethnicity**	228		9004		<.001
Hispanic		36 (15.3)		1961 (16.0)	
Non-Hispanic Black		104 (28.8)		2355 (11.2)	
Non-Hispanic White		59 (47.4)		3158 (62.8)	
Other[b]		29 (8.55)		1530 (10.0)	
**Educational attainment**	226		8991		<.001
Less than high school graduate		56 (21.2)		1704 (10.9)	

© 2022 Di H et al. *JAMA Network Open.*

High school graduate or GED		79 (44.3)		2146 (26.7)	
Some college or above		91 (32.6)		5141 (62.4)	
**Family income level to poverty level**	163		7665		<.001
<1.30		67 (24.2)		2135 (16.4)	
1.30-3.49		66 (32.0)		3021 (30.5)	
≥3.50		30 (11.8)		1339 (40.7)	
**Work status**	227		8994		<.001
Non-employed		131 (55.9)		3996 (37.2)	
Part-time (1–34 hours/week)		29 (11.2)		1292 (15.6)	
Full-time (≥ 35 hours/week)		67 (32.9)		3706 (47.3)	
**Work schedule**	228		9004		<.001
Traditional 9 AM to 5 PM day		21 (8.31)		1972 (26.7)	
Regular shift work		32 (17.5)		1383 (14.5)	
Rotating shift work		46 (20.4)		1774 (23.3)	
Did not work		129 (53.8)		3875 (35.5)	

[a] Unweighted number of participants. All percentage estimates were weighted.

[b] “Other” includes race/ethnicity other than non-Hispanic White, non-Hispanic black, or Hispanic.

© 2022 Di H et al. *JAMA Network Open.*

eTable 3. Estimated Means and Distributions of Sleep Duration During Work Days and Free Days Among US Adults Aged 20 Years or Older, NHANES 2017-2020[a]

Characteristics	Sleep duration, Mean (95% CI)		*P* value[b]	<7 h, % (95% CI)		*P* value[b]	7–9 h, % (95% CI)		*P* value[b]	≥9 h, % (95% CI)		*P* value[b]
	**Work days**	**Free days**		**Work days**	**Free days**		**Work days**	**Free days**		**Work days**	**Free days**	
**Overall**	7.59 (7.54 - 7.64)	8.24 (8.17 - 8.31)	<.001	23.1 (21.3 - 24.9)	12.9 (11.6 - 14.1)	<.001	57.2 (54.8 - 59.6)	48.7 (46.9 - 50.4)	<.001	19.7 (18.5 - 21.0)	38.5 (36.7 - 40.3)	<.001
**Age (years)**												
20-39	7.63 (7.58 - 7.69)	8.45 (8.35 -8.54)	<.001	22.3 (20.3 - 24.2)	10.3 (8.6 - 12.1)	<.001	57.3 (54.1 - 60.5)	44.5 (42.3 - 46.6)	<.001	20.5 (18.4 - 22.5)	45.2 (42.5 - 47.9)	<.001
40-59	7.37 (7.28 - 7.46)	8.20 (8.11 -8.29)	<.001	26.9 (23.9 - 29.8)	13.6 (11.7 - 15.5)	<.001	59.6 (56.2 - 62.9)	48.9 (45.7 - 52.0)	<.001	13.6 (11.5 - 15.6)	37.5 (34.6 - 40.4)	<.001
60-74	7.66 (7.53 - 7.80)	7.96 (7.84 -8.08)	<.001	21.8 (18.2 - 25.5)	15.9 (13.2 - 18.7)	<.001	56.2 (52.7 - 59.6)	54.6 (51.3 - 57.9)	.71	22.0 (19.2 - 24.8)	29.5 (26.8 - 32.2)	<.001
≥75	8.15 (8.00 - 8.31)	8.22 (8.07 -8.37)	.02	14.6 (11.3 - 17.9)	12.7 (9.9 - 15.5)	.04	49.8 (45.6 - 54.1)	50.9 (46.9 - 54.9)	.49	35.6 (31.2 - 39.9)	36.4 (31.9 - 40.9)	.38
**Sex**												
Female	7.74 (7.68 - 7.81)	8.39 (8.31 -8.47)	<.001	19.6 (17.4 - 21.7)	10.9 (9.4 - 12.3)	<.001	57.1 (54.2 - 59.9)	46.3 (44.0 - 48.7)	<.001	23.4 (21.5 - 25.2)	42.8 (40.6 - 45.0)	<.001
Male	7.43 (7.39 - 7.48)	8.07 (8.00 -8.15)	<.001	26.9 (24.9 - 28.9)	15.0 (13.4 - 16.6)	<.001	57.3 (54.6 - 60.1)	51.2 (49.2 - 53.2)	<.001	15.8 (14.4 - 17.1)	33.8 (31.6 - 36.0)	<.001
**Race/ethnicity**												

© 2022 Di H et al. *JAMA Network Open.*

Hispanic	7.59 (7.49 - 7.70)	8.56 (8.41 -8.70)	<.001	25.6 (22.8 - 28.4)	11.9 (9.8 - 14.1)	<.001	53.2 (50.4 - 56.0)	38.9 (36.3 - 41.5)	<.001	21.2 (18.4 - 24.0)	49.2 (45.7 - 52.7)	<.001
Non-Hispanic Black	7.42 (7.35 - 7.49)	8.12 (8.02 -8.22)	<.001	32.0 (29.2 - 34.7)	21.4 (19.4 - 23.4)	<.001	45.1 (41.7 - 48.5)	39.2 (36.5 - 41.9)	<.001	23.0 (21.0 - 24.9)	39.4 (36.9 - 41.9)	<.001
.Non-Hispanic White	7.63 (7.58 - 7.69)	8.18 (8.11 -8.25)	<.001	20.7 (18.4 - 23.1)	11.5 (9.7 - 13.4)	<.001	60.5 (57.3 - 63.8)	53.0 (50.6 - 55.5)	<.001	18.7 (17.2 - 20.2)	35.4 (33.4 - 37.5)	<.001
Other[c]	7.55 (7.45 - 7.66)	8.25 (8.15 -8.35)	<.001	24.0 (20.3 - 27.6)	13.0 (10.0 - 16.0)	<.001	56.2 (51.4 - 61.0)	47.6 (43.5 - 51.6)	<.001	19.9 (16.9 - 22.9)	39.4 (37.1 - 41.8)	<.001
**Educational attainment**												
<High school	7.72 (7.59 - 7.84)	8.46 (8.31 -8.60)	<.001	25.8 (22.3 - 29.4)	15.2 (11.9 - 18.5)	<.001	47.1 (43.5 - 50.6)	38.3 (34.9 - 41.8)	<.001	27.1 (23.6 - 30.6)	46.4 (42.7 - 50.2)	<.001
High school	7.63 (7.54 - 7.72)	8.22 (8.09 -8.35)	<.001	24.0 (21.2 - 26.8)	15.6 (13.2 - 18.0)	<.001	52.5 (49.3 - 55.7)	42.7 (39.9 - 45.4)	<.001	23.5 (20.9 - 26.1)	41.7 (38.6 - 44.8)	<.001
>High school	7.56 (7.49 - 7.62)	8.21 (8.15 -8.27)	<.001	22.2 (19.9 - 24.6)	11.3 (9.7 - 12.9)	<.001	61.0 (58.0 - 63.9)	53.0 (51.1 - 55.0)	<.001	16.8 (15.4 - 18.2)	35.7 (33.8 - 37.6)	<.001
**Family income level (IPR)**												
<1.30	7.68 (7.56 - 7.79)	8.25 (8.12 -8.38)	<.001	25.5 (22.3 - 28.6)	17.8 (15.1 - 20.5)	<.001	45.5 (42.3 - 48.8)	38.5 (35.6 - 41.4)	<.001	29.0 (26.6 - 31.4)	43.7 (40.6 - 46.9)	<.001
1.30-3.49	7.63 (7.55 - 7.71)	8.28 (8.19 -8.37)	<.001	24.2 (21.2 - 27.2)	14.4 (12.4 - 16.5)	<.001	54.2 (50.7 - 57.8)	44.4 (41.1 - 47.6)	<.001	21.5 (19.3 - 23.8)	41.2 (38.6 - 43.8)	<.001

© 2022 Di H et al. *JAMA Network Open.*

≥3.50	7.51 (7.41 - 7.62)	8.21 (8.13 -8.28)	<.001	21.5 (18.4 - 24.7)	9.23 (7.04 - 11.5)	<.001	65.0 (61.6 - 68.4)	56.6 (54.1 - 59.1)	<.001	13.5 (11.6 - 15.3)	34.2 (31.8 - 36.5)	<.001
**Work status**												
Non-employed	7.95 (7.87 - 8.13)	8.20 (8.10 - 8.35)	<.001	19.3 (17.3 - 21.2)	15.1 (13.2 - 16.9)	<.001	49.1 (46.5 - 51.8)	47.3 (44.5 - 50.1)	.05	31.6 (29.7 - 33.5)	37.6 (35.4 - 39.8)	<.001
Part-time (1–34 hours/week)	7.73 (7.63 - 7.82)	8.29 (8.16 - 8.41)	<.001	18.8 (15.2 - 22.4)	10.7 (8.2 - 13.1)	<.001	60.1 (54.8 - 65.3)	51.4 (45.8 - 57.1)	.01	21.1 (17.8 - 24.4)	37.9 (32.4 - 43.4)	<.001
Full-time (≥ 35 hours/week)	7.27 (7.21 - 7.33)	8.25 (8.17 - 8.33)	<.001	27.5 (25.1 - 29.9)	11.8 (10.0 - 13.6)	<.001	62.6 (59.8 - 65.3)	48.8 (46.6 - 51.0)	<.001	9.87 (8.85 - 10.9)	39.4 (37.1 - 41.7)	<.001
**Work schedule**												
Traditional 9 AM to 5 PM day	7.53 (7.46 - 7.62)	8.29 (8.19 - 8.38)	<.001	18.5 (15.8 - 21.1)	8.90 (6.46 - 11.3)	<.001	70.6 (67.2 - 74.0)	52.5 (49.5 - 55.5)	<.001	10.9 (9.2 - 12.7)	38.6 (35.6 - 41.6)	<.001
Regular shift work	7.14 (7.73 - 7.26)	8.45 (8.32 - 8.59)	<.001	37.0 (31.9 - 42.0)	12.2 (10.0 - 14.5)	<.001	49.1 (44.0 - 54.3)	41.3 (37.5 - 45.1)	.004	13.9 (11.5 - 16.3)	46.4 (42.1 - 50.8)	<.001
Rotating shift work	7.36 (7.26 - 7.47)	8.10 (7.98 - 8.21)	<.001	25.9 (22.0 - 29.8)	14.1 (11.4 - 16.8)	<.001	60.3 (56.3 - 64.4)	52.0 (48.2 - 55.8)	<.001	13.8 (11.1 - 16.5)	34.0 (30.3 - 37.7)	<.001
Did not work	7.98 (7.89 - 8.46)	8.21 (8.11 - 8.31)	<.001	19.1 (17.2 - 21.0)	15.3 (13.5 - 17.1)	<.001	48.4 (45.8 - 50.9)	46.7 (43.8 - 49.5)	.07	32.6 (30.7 - 34.4)	38.1 (35.7 - 40.4)	<.001

[a] Weighted estimates and 95% CIs were estimated for each stratums. All estimates were weighted to be nationally representative.

[b] P value for differences between weekday and weekend of each subgroup.

[c] “Other” includes race/ethnicity other than non-Hispanic White, non-Hispanic Black, or Hispanic.

© 2022 Di H et al. *JAMA Network Open.*

eTable 4. Estimated Means and Distributions of Usual Sleep Time During Work Days and Free Days Among US Adults Aged 20 Years or Older, NHANES 2017-2020[a]

Characteristics	Sleep time, Mean (95% CI)		*P* value[b]	Before 22:00, % (95% CI)		*P* value[b]	22:00 to midnight, % (95% CI)		*P* value[b]	Midnight or later, % (95% CI)		*P* value[b]
	**Work days**	**Free days**		**Workdays**	**Free days**		**Work days**	**Free days**		**Work days**	**Free days**	
**Overall**	23:02 (22:57 - 23:17)	23:25 (23:21 - 23:35)	<.001	18.5 (16.8 - 20.2)	11.0 (9.70 - 12.3)	<.001	56.1 (54.4 - 57.8)	48.1 (46.0 - 50.1)	<.001	25.4 (24.1 - 26.6)	40.9 (38.4 - 43.5)	<.001
**Age (years)**												
20-39	23:26 (23:14 - 23:38)	00:01 (23:56 - 00:06)	<.001	14.5 (12.3 - 16.7)	5.62 (4.71 - 6.53)	<.001	52.9 (50.1 - 55.7)	38.1 (35.4 - 40.8)	<.001	32.6 (30.2 - 35.1)	56.3 (53.3 - 59.2)	<.001
40-59	22:52 (22:45 - 22:62)	23:17 (23:12 - 23:23)	<.001	20.1 (17.6 - 22.7)	11.5 (9.5 - 13.5)	<.001	58.7 (55.9 - 61.5)	50.0 (46.6 - 53.5)	<.001	21.2 (18.9 - 23.5)	38.5 (35.2 - 41.8)	<.001
60-74	22:48 (22:42 - 22:54)	22:57 (22:50 - 23:03)	.007	22.0 (18.6 - 25.3)	16.2 (12.9 - 19.5)	<.001	56.5 (52.8 - 60.1)	57.5 (53.8 - 61.2)	<.001	21.6 (19.2 - 24.0)	26.3 (23.6 - 29.0)	<.001
≥75	22:37 (22:29 - 22:46)	22:41 (22:33 - 22:49)	<.001	20.4 (17.3 - 23.5)	18.8 (16.0 - 21.6)	<.001	58.9 (56.1 - 61.8)	59.1 (56.3 - 61.9)	.46	20. 7 (17.9 - 23.4)	22.1 (19.2 - 25.0)	.01
**Sex**												
Female	22:54 (22:48 - 23:41)	23:18 (23:12 - 23:23)	<.001	19.5 (17.4 - 21.6)	11.8 (10.0 - 13.6)	<.001	56.9 (55.0 - 58.8)	51.4 (49.4 - 53.4)	<.001	23.6 (22.0 - 25.2)	36.8 (34.2 - 39.4)	<.001
Male	23:10 (23:52 - 23:19)	23:34 (23:28 - 23:40)	<.001	17.5 (15.5 - 19.4)	10.1 (8.4 - 11.8)	<.001	55.3 (53.1 - 57.5)	44.5 (41.2 - 47.8)	<.001	27.3 (25.0 - 29.5)	45.4 (41.9 - 48.8)	<.001
**Race/ethnicity**												

© 2022 Di H et al. *JAMA Network Open.*

Hispanic	22:54 (22:44 - 23:66)	23:26 (23:19 - 23:34)	<.001	20.8 (16.9 - 24.7)	10.0 (8.0 - 12.1)	<.001	53.6 (50.6 - 56.7)	48.4 (45.3 - 51.4)	.008	25.6 (22.0 - 29.2)	41.6 (37.7 - 45.5)	<.001
Non-Hispanic Black	23:18 (23:15 - 23:27)	23:45 (23:38 - 23:52)	<.001	21.2 (19.1 - 23.4)	13.3 (11.5 - 15.0)	<.001	46.3 (43.9 - 48.8)	36.3 (34.1 - 38.5)	<.001	32.4 (30.3 - 34.6)	50.4 (48.0 - 52.8)	<.001
Non-Hispanic White	22:59 (22:53 - 23:36)	23:20 (23:14 - 23:26)	<.001	18.5 (16.0 - 20.9)	11.4 (9.4 - 13.3)	<.001	58.6 (56.1 - 61.2)	50.8 (48.2 - 53.3)	<.001	22.9 (21.3 - 24.5)	37.9 (34.8 - 41.0)	<.001
Other[c]	23:14 (23:06 - 23:22)	23:39 (23:33 - 23:45)	<.001	12.2 (9.7 - 14.6)	7.74 (5.56 - 9.91)	<.001	55.3 (50.9 - 59.6)	43.9 (40.5 - 47.2)	<.001	32.6 (28.9 - 36.3)	48.4 (45.2 - 51.6)	<.001
**Educational attainment**												
<High school	22:46 (22:37 - 22:56)	23:12 (23:04 - 23:20)	<.001	24.4 (21.5 - 27.3)	16.8 (14.0 - 19.7)	<.001	50.0 (46.2 - 53.8)	46.4 (42.6 - 50.3)	.02	25.6 (22.0 - 29.2)	36.7 (33.2 - 40.3)	<.001
High school	23:03 (22:50 - 23:17)	23:23 (23:16 - 23:31)	.002	22.6 (18.7 - 26.6)	13.7 (11.5 - 15.9)	<.001	49.7 (46.2 - 53.1)	45.9 (42.7 - 49.1)	.01	27.7 (24.8 - 30.6)	40.4 (37.6 - 43.2)	<.001
>High school	23:04 (22:59 - 23:10)	23:29 (23:24 - 23:34)	<.001	15.7 (13.4 - 18.0)	8.79 (7.32 - 10.3)	<.001	59.9 (57.7 - 62.2)	49.3 (46.5 - 52.0)	<.001	24.3 (22.7 - 26.0)	41.9 (38.7 - 45.1)	<.001
**Family income level (IPR)**												
<1.30	23:10 (23:01 - 23:19)	23:40 (23:30 - 23:50)	<.001	21.4 (18.7 - 24.1)	12.2 (10.3 - 14.0)	<.001	43.6 (40.0 - 47.2)	39.5 (35.6 - 43.4)	.002	34.9 (30.8 - 39.0)	48.4 (44.2 - 52.6)	<.001
1.30-3.49	23:07 (22:56 - 23:19)	23:28 (23:21 - 23:36)	<.001	18.7 (16.5 - 20.8)	12.0 (10.6 - 13.4)	<.001	53.4 (50.5 - 56.2)	44.9 (41.8 - 47.9)	<.001	28.0 (25.5 - 30.4)	43.1 (39.8 - 46.4)	<.001
≥3.50	22:55 (22:48 - 23:03)	23:18 (23:11 - 23:24)	<.001	17.3 (14.6 - 19.9)	9.75 (7.83 - 11.7)	<.001	62.4 (59.6 - 65.3)	53.1 (49.8 - 56.5)	<.001	20.3 (18.0 - 22.6)	37.1 (33.4 - 40.9)	<.001
**Work status**												

© 2022 Di H et al. *JAMA Network Open.*

Non-employed	22:56 (22:51-23:02)	23:16 (23:10-23:21)	<.001	18.1 (16.1 - 20.2)	13.4 (11.8 - 15.0)	<.001	54.2 (52.2 - 56.2)	50.0 (47.1 - 52.9)	<.001	27.7 (25.9 - 29.5)	36.6 (33.5 - 39.6)	<.001
Part-time (1–34 hours/week)	23:15 (23:03-23:27)	23:39 (23:29-23:50)	<.001	13.8 (11.0 - 16.5)	8.42 (5.91 - 10.9)	<.001	57.0 (53.0 - 60.9)	44.7 (40.7 - 48.7)	<.001	29.3 (24.8 - 33.7)	46.9 (41.8 - 52.0)	<.001
Full-time (≥ 35 hours/week)	23:03 (22:55-23:11)	23:29 (23:23-23:35)	<.001	20.3 (18.0 - 22.7)	9.86 (8.17 - 11.5)	<.001	57.4 (55.2 - 59.6)	47.7 (45.3 - 50.1)	<.001	22.3 (20.2 - 24.3)	42.4 (39.3 - 45.6)	<.001
**Work schedule**												
Traditional 9 AM to 5 PM day	22:36 (22:31-22:41)	23:22 (23:16-23:28)	<.001	18.2 (15.2 - 21.2)	7.81 (5.85 - 9.77)	<.001	67.5 (64.4 - 70.7)	52.0 (48.5 - 55.6)	<.001	14.3 (12.5 - 16.1)	40.2 (36.5 - 43.8)	<.001
Evening or nights	02:46 (02:09-02:82)	0:33 (0:05-1:01)	<.001	9.32 (6.06 - 12.6)	10.4 (6.1 - 14.7)	.60	18.0 (12.7 - 23.4)	26.9 (23.3 - 30.6)	<.001	72.7 (66.8 - 78.5)	62.7 (56.9 - 68.5)	.002
Early morning	22:02 (21:49-22:16)	22:54 (22:43-23:04)	<.001	38.3 (32.9 - 43.7)	17.0 (12.8 - 21.1)	<.001	51.8 (46.4 - 57.1)	51.9 (46.3 - 57.5)	.97	9.95 (6.15 - 13.7)	31.2 (25.2 - 37.1)	<.001
Rotating shift work	23:04 (22:57-23:12)	23:39 (23:30-23:49)	<.001	14.4 (11.7 - 17.2)	8.27 (6.02 - 10.5)	<.001	58.2 (55.5 - 61.0)	44.7 (41.0 - 48.5)	<.001	27.3 (24.4 - 30.3)	47.0 (42.6 - 51.4)	<.001
Did not work	22:56 (22:50-23:01)	23:15 (23:09-23:21)	<.001	18.4 (16.4 - 20.4)	13.9 (12.2 - 15.5)	<.001	53.8 (51.5 to 56.0)	50.0 (47.2 to 52.9)	<.001	27.9 (25.9 to 29.8)	36.1 (33.0 to 39.3)	<.001

[a] Weighted estimates and 95% CIs were estimated for each stratums. All estimates were weighted to be nationally representative.

© 2022 Di H et al. *JAMA Network Open.*

[b] P value for differences between weekday and weekend of each subgroup.

[c] “Other” includes race/ethnicity other than non-Hispanic White, non-Hispanic Black, or Hispanic.

© 2022 Di H et al. *JAMA Network Open.*

eTable 5. Estimated Means and Distributions of Usual Wake Time During Work Days and Free Days Among US Adults Aged 20 Years or Older, NHANES 2017-2020[a]

Characteristics	Wake time, Mean (95% CI)		*P* value[b]	Before 6:00, % (95% CI)		*P* value[b]	6:00 to 8:00, % (95% CI)		*P* value[b]	8:00 or later, % (95% CI)		*P* value[b]
	Work days	Free days		Workdays	Free days		Work days	Free days		Work days	Free days	
**Overall**	6:41 (6:36 - 6:45)	7:41 (7:37 - 7:46)	<.001	31.6 (29.8 - 33.5)	12.0 (10.7 - 13.4)	<.001	47.0 (44.9 - 49.1)	40.3 (38.7 - 41.9)	<.001	21.4 (20.1 - 22.6)	47.7 (45.6 - 49.7)	<.001
**Age (years)**												
20-39	7:07 (6:55 - 7:19)	8:30 (8:24 - 8:36)	<.001	25.8 (23.4 - 28.2)	4.27 (3.30 - 5.24)	<.001	46.4 (42.5 - 50.3)	30.3 (28.2 - 32.4)	<.001	27.8 (24.9 - 30.8)	65.4 (63.1 - 67.8)	<.001
40-59	6:18 (6:11 - 6:25)	7:30 (7:24 - 7:36)	<.001	39.3 (35.8 - 42.7)	12.1 (9.9 - 14.2)	<.001	46.9 (42.8 - 51.1)	43.4 (40.4 - 46.5)	.11	13.8 (11.7 - 15.9)	44.5 (41.4 - 47.7)	<.001
60-74	6:29 (6:21 - 6:37)	6:56 (6:48 - 7:03)	<.001	33.3 (29.4 - 37.3)	22.7 (19.0 - 26.4)	<.001	46.0 (42.5 - 49.5)	47.5 (43.9 - 51.0)	.33	20.7 (17.4 - 23.9)	29.9 (26.8 - 33.0)	<.001
≥75	6:47 (6:40 - 6:53)	6:58 (6:49 - 7:07)	<.001	21.4 (18.4 - 24.5)	18.0 (14.3 - 21.7)	<.001	52.3 (47.5 - 57.1)	52.6 (47.6 - 57.7)	.75	26.3 (21.8 - 30.8)	29.3 (24.9 - 33.7)	.003
**Sex**												
Female	6:42 (6:37 - 6:48)	7:42 (7:37 - 7:47)	<.001	29.1 (27.1 - 31.1)	11.2 (9.6 - 12.7)	<.001	48.8 (46.2 - 51.4)	40.3 (37.7 - 43.0)	<.001	22.1 (20.1 - 24.1)	48.5 (46.1 - 50.9)	<.001
Male	6:39 (6:29 - 6:48)	7:41 (7:35 - 7:47)	<.001	34.3 (32.0 - 36.6)	12.9 (11.2 - 14.7)	<.001	45.1 (42.0 - 48.2)	40.3 (38.1 - 42.5)	.008	20.6 (18.4 - 22.9)	46.7 (44.3 - 49.2)	<.001
**Race/ethnicity**												

© 2022 Di H et al. *JAMA Network Open.*

Hispanic	6:32 (6:21 - 6:43)	7:59 (7:52 - 8:06)	<.001	34.3 (30.5 - 38.2)	9.08 (7.68 - 10.5)	<.001	45.9 (43.4 - 48.5)	33.8 (31.1 - 36.5)	<.001	19.7 (16.9 - 22.6)	57.1 (54.0 - 60.2)	<.001
Non-Hispanic Black	6:48 (6:40 - 6:56)	7:55 (7:48 - 8:03)	<.001	33.8 (31.7 - 35.9)	13.6 (11.8 - 15.4)	<.001	42.4 (39.7 - 45.0)	33.8 (32.1 - 35.4)	<.001	23.8 (21.7 - 25.9)	52.7 (50.3 - 55.0)	<.001
Non-Hispanic White	6:40 (6:33 - 6:48)	7:32 (7:27 - 7:38)	<.001	31.5 (28.7 - 34.3)	12.9 (11.0 - 14.8)	<.001	47.6 (44.9 - 50.3)	44.0 (41.9 - 46.1)	.02	20.9 (19.4 - 22.5)	43.1 (40.7 - 45.6)	<.001
Other[c]	6:50 (6:41 - 6:58)	7:54 (7:48 - 8:01)	<.001	25.6 (22.3 - 28.9)	9.57 (7.57 - 11.6)	<.001	50.3 (46.5 - 54.0)	34.9 (31.9 - 37.8)	<.001	24.1 (21.3 - 26.9)	55.6 (52.4 - 58.7)	<.001
**Educational attainment**												
<High school	6:34 (6:21 - 6:47)	7:43 (7:33 - 7:53)	<.001	36.9 (31.8 - 42.0)	14.7 (12.2 - 17.3)	<.001	40.0 (36.4 - 43.6)	36.9 (33.8 - 40.0)	.12	23.1 (20.1 - 26.1)	48.4 (44.7 - 52.0)	<.001
High school	6:43 (6:31 - 6:55)	7:40 (7:32 - 7:49)	<.001	35.2 (31.8 - 38.6)	15.2 (12.2 - 18.3)	<.001	40.2 (38.1 - 42.2)	37.3 (34.1 - 40.6)	.05	24.6 (22.0 - 27.2)	47.4 (44.1 - 50.8)	<.001
>High school	6:41 (6:36 - 6:46)	7:42 (7:37 - 7:47)	<.001	29.2 (27.1 - 31.2)	10.2 (8.6 - 11.8)	<.001	51.1 (48.7 - 53.5)	42.2 (40.1 - 44.2)	<.001	19.7 (18.2 - 21.3)	47.7 (45.3 - 50.1)	<.001
**Family income level (IPR)**												
<1.30	6:54 (6:46 - 7:03)	7:55 (7:47 - 8:03)	<.001	28.5 (25.4 - 31.7)	13.2 (11.3 - 15.1)	<.001	41.8 (38.8 - 44.9)	33.7 (29.3 - 38.2)	<.001	29.6 (26.5 - 32.8)	53.1 (48.9 - 57.2)	<.001
1.30-3.49	6:51 (6:39 - 7:03)	7:49 (7:40 - 7:58)	<.001	31.1 (27.9 - 34.3)	11.9 (9.6 - 14.1)	<.001	43.4 (40.2 - 46.6)	36.9 (33.5 - 40.2)	<.001	25.5 (22.7 - 28.3)	51.3 (48.2 - 54.4)	<.001
≥3.50	6:27 (6:19 - 6:35)	7:31 (7:25 - 7:38)	<.001	33.8 (30.1 - 37.5)	11.5 (9.6 - 13.3)	<.001	51.4 (47.7 - 55.1)	45.8 (43.0 - 48.7)	.01	14.8 (12.8 - 16.8)	42.7 (39.3 - 46.2)	<.001
**Work status**												

© 2022 Di H et al. *JAMA Network Open.*

Non-employed	6:55 (6:50-6:60)	7:29 (7:22-7:36)	<.001	22.5 (20.5 - 24.6)	15.2 (13.6 - 16.7)	<.001	48.6 (46.7 - 50.6)	41.7 (38.5 - 44.8)	<.001	28.8 (26.9 - 30.8)	43.2 (40.3 - 46.0)	<.001
Part-time (1–34 hours/week)	6:58 (6:45-7:50)	7:58 (7:45-8:11)	<.001	22.3 (18.0 - 26.6)	9.24 (6.30 - 12.2)	<.001	51.5 (47.1 - 55.9)	38.6 (33.9 - 43.4)	<.001	26.2 (22.7 - 29.7)	52.1 (46.8 - 57.5)	<.001
Full-time (≥ 35 hours/week)	6:24 (6:16-6:32)	7:46 (7:41-7:52)	<.001	41.9 (39.3 - 44.4)	10.4 (8.6 - 12.3)	<.001	44.2 (41.2 - 47.2)	39.8 (37.8 - 41.8)	.01	14.0 (12.2 - 15.7)	49.8 (47.3 - 52.3)	<.001
**Work schedule**												
Traditional 9 AM to 5 PM day	6:06 (6:02-6:11)	7:39 (7:32-7:47)	<.001	34.0 (30.3 - 37.8)	8.79 (6.80 - 10.8)	<.001	61.6 (57.9 - 65.3)	43.0 (39.8 - 46.2)	<.001	4.36 (3.38 - 5.35)	48.2 (44.7 - 51.7)	<.001
Evening or nights	10:6 (10:1-11:4)	9:22 (9:01-9:43)	<.001	5.05 (3.40 - 6.70)	4.22 (2.03 - 6.40)	.50	18.5 (13.9 - 23.1)	24.3 (19.6 - 28.9)	.02	76.4 (71.4 - 81.5)	71.5 (66.5 - 76.5)	.09
Early morning	4:56 (4:42-5:09)	7:20 (7:09-7:30)	<.001	80.6 (76.1 - 85.0)	16.3 (12.5 - 20.1)	<.001	15.9 (11.3 - 20.6)	41.6 (36.8 - 46.4)	<.001	3.5 (1.4 - 5.6)	42.1 (36.6 - 47.7)	<.001
Rotating shift work	6:28 (6:20-6:35)	7:45 (7:37-7:54)	<.001	33.0 (29.3 - 36.8)	10.9 (8.2 - 13.5)	<.001	46.6 (43.2 - 50.0)	38.9 (35.3 - 42.5)	<.001	20.3 (18.3 - 22.3)	50.2 (46.5 - 54.0)	<.001
Did not work	6:56 (6:51-7:01)	7:29 (7:21-7:36)	<.001	21.9 (19.9 - 23.9)	15.5 (13.9 - 17.1)	<.001	48.5 (46.5 to 50.5)	41.7 (38.4 to 45.0)	<.001	29.6 (27.6 to 31.6)	42.8 (39.8 to 45.8)	<.001

[a] Weighted estimates and 95% CIs were estimated for each stratums. All estimates were weighted to be nationally representative.

[b] P value for differences between weekday and weekend of each subgroup.

[c] “Other” includes race/ethnicity other than non-Hispanic White, non-Hispanic Black, or Hispanic.

© 2022 Di H et al. *JAMA Network Open.*

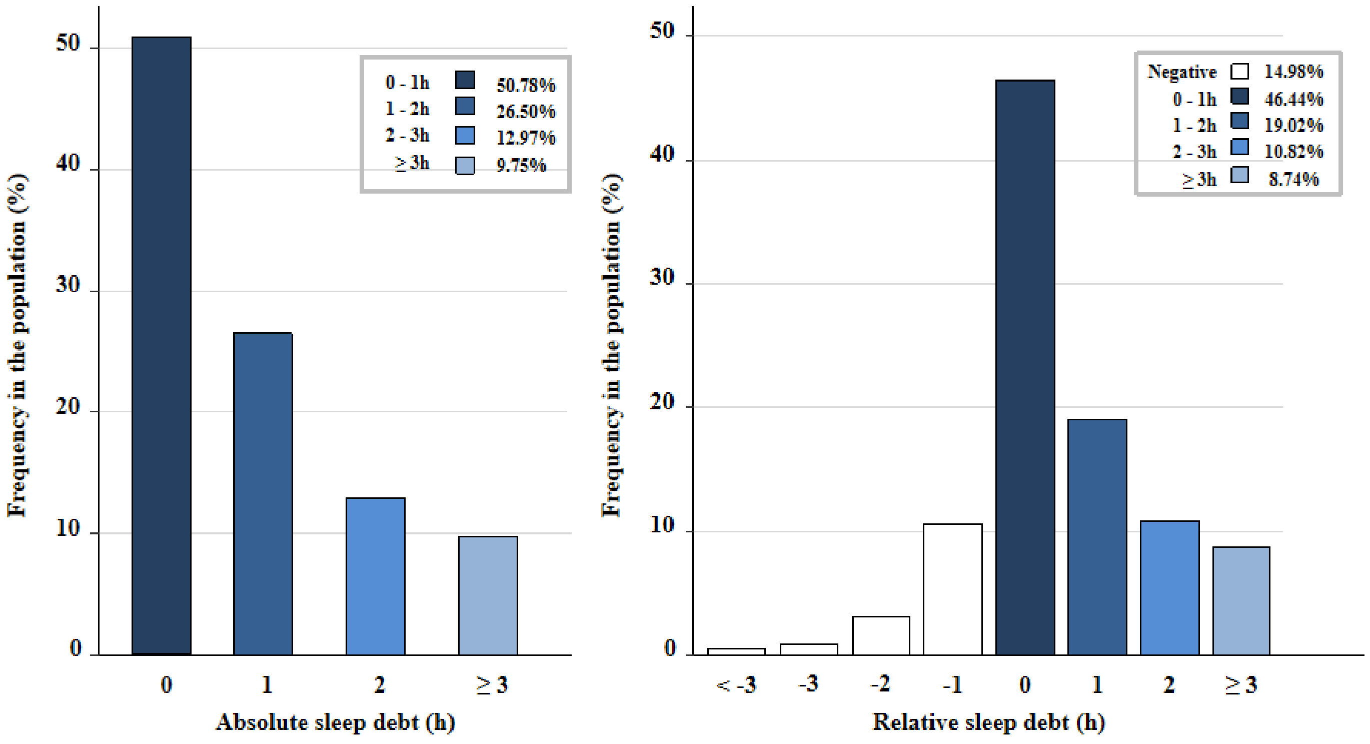

eFigure 1. Distributions of Sleep Debt (Absolute Difference in Sleep Duration Between Work and Free Days)

Absolute sleep debt (left panel) and relative sleep debt (right panel) are shown. The distribution is based on one-hourly bins, except for the categories at both ends. The lowest bins of sleep debt represent <1 h (left panel) and <-3 h (right panel); The highest bins of sleep debt represent ≥3 h; intermediary bins include the lower and exclude the upper limit.

© 2022 Di H et al. *JAMA Network Open.*

eTable 6. Estimated Means and Distributions of Sleep Debt (Absolute Difference Between the Average Weekday and Free-Day Sleep Duration) Among US Adults Aged 20 Years or Older, NHANES 2017-2020[a]

Characteristics	Sleep debt Mean (95% CI)	*P* value[b]	0-1h, % (95% CI)	*P* value[b]	1-2h % (95% CI)	*P* value[b]	≥ 2h % (95% CI)	*P* value[b]
**Overall.[c]**	0.73 (0.68 - 0.77)		70.0 (67.6 - 72.3)		20.3 (18.7 - 22.0)		9.75 (8.65 - 10.8)	
**Age (years)**								
20-39	0.92 (0.87 -0.97)	<.001	61.2 (58.2 - 64.2)	<.001	26.1 (23.6 - 28.6)	<.001	12.8 (11.2 - 14.3)	<.001
40-59	0.83 (0.77 -0.89)		64.7 (61.4 - 68.0)		23.3 (21.0 - 25.5)		12.0 (9.64 - 14.5)	
60-64	0.55 (0.47 - 0.62)		77.8 (73.5 - 82.2)		16.9 (12.9 - 20.8)		5.26 (3.23 - 7.29)	
65-74	0.32 (0.28 - 0.36)		89.3 (87.2 - 91.3)		7.69 (5.52 - 9.85)		3.06 (2.07 - 4.05)	
≥75	0.24 (0.18 - 0.30)		91.7 (89.0 - 94.5)		6.03 (4.01 to 8.04)		2.23 (0.95 - 3.51)	
**Sex**								
Female	0.71 (0.66 -0.75)	.09	71.3 (68.7 - 74.0)	.06	19.8 (17.7 - 21.9)	.38	8.89 (7.67 - 10.1)	.02
Male	0.75 (0.69 -0.80)		68.5 (65.5 - 71.5)		20.9 (18.8 - 23.0)		10.7 (9.10 - 12.2)	
**Race/ethnicity**								
Hispanic	0.97 (0.92 -1.02)	<.001	57.9 (55.2 - 60.5)	<.001	26.5 (23.3 - 29.6)		15.7 (13.5 - 17.8)	<.001
Non-Hispanic Black	0.97 (0.91 -1.02)		60.5 (57.9 - 63.0)		23.8 (21.8 - 25.8)	<.001	15.7 (13.8 - 17.7)	
Non-Hispanic White	0.62 (0.57 -0.68)		74.6 (71.3 - 78.0)		18.0 (15.5 - 20.6)		7.33 (5.77 - 8.90)	

© 2022 Di H et al. *JAMA Network Open.*

Other[d]	0.71 (0.65 -0.77)		70.4 (66.7 - 74.2)		20.8 (18.0 - 23.6)		8.77 (6.31 - 11.2)	
**Educational attainment**								
<High school	0.82 (0.72 -0.92)	.003	66.9 (62.5 - 71.2)	.006	18.6 (15.6 - 21.5)	.51	14.6 (11.7 - 17.5)	<.001
High school	0.77 (0.69 -0.86)		67.5 (63.3 - 71.7)		20.5 (17.9 - 23.2)		11.9 (9.00 - 14.8)	
>High school	0.69 (0.66 -0.72)		71.5 (69.5 - 73.6)		20.5 (18.6 - 22.4)		7.97 (7.21 - 8.73)	
**Family income level (IPR)**								
<1.30	0.76 (0.70 -0.83)	.04	69.0 (66.0 - 71.9)	.28	18.5 (15.6 - 21.4)	.17	12.5 (9.81 - 15.2)	<.001
1.30-3.49	0.77 (0.70 -0.85)		68.3 (65.1 - 71.5)		19.5 (17.2 - 21.8)		12.2 (10.2 - 14.2)	
≥3.50	0.68 (0.62 -0.73)		71.3 (67.9 - 74.7)		21.9 (19.0 - 24.9)		6.76 (5.45 - 8.07)	
**Work status**								
Non-employed	0.43 (0.39 - 0.47)	<.001	83.0 (80.8 - 85.2)	<.001	12.5 (10.4 - 14.6)	<.001	4.51 (3.59 - 5.43)	<.001
Part-time (1–34 hours/week)	0.69 (0.62 - 0.76)		72.2 (67.9 - 76.6)		19.0 (15.3 - 22.6)		8.81 (6.77 - 10.8)	
Full-time (≥ 35 hours/week)	0.97 (0.91 - 1.03)		58.9 (55.8 - 62.0)		26.9 (24.6 - 29.2)		14.2 (12.5 - 15.9)	
**Work schedule**								
Traditional 9 AM to 5 PM day	0.78 (0.72 - 0.84)	<.001	66.7 (63.6 - 69.7)	<.001	25.5 (23.4 - 27.6)	<.001	7.84 (6.00 - 9.68)	<.001
Regular shift work	1.23 (1.13 - 1.33)		48.4 (42.7 - 54.2)		28.8 (24.4 - 33.3)		22.7 (20.0 - 25.4)	
Rotating shift work	0.82 (0.75 - 0.88)		66.3 (62.3 - 70.3)		21.7 (18.2 - 25.1)		12.0 (9.0 - 15.1)	
Did not work	0.42 (0.38 - 0.46)		83.6 (81.4 - 85.8)		12.0 (10.0 - 14.0)		4.40 (3.50 - 5.30)	

[a] Weighted estimates and 95% CIs were estimated for each stratums. All estimates were weighted to be nationally representative.

[b] P value for overall differences across stratums.

© 2022 Di H et al. *JAMA Network Open.*

[c] Sleep debt was calculated as the absolute difference between sleep duration (duration from sleep onset time until wake time) on free days minus the weekly average sleep duration (approximated by averaging 5 work and 2 free days).

[d] “Other” includes race/ethnicity other than non-Hispanic White, non-Hispanic Black, or Hispanic.

© 2022 Di H et al. *JAMA Network Open.*

eTable 7. Estimated Means and Distributions of Sleep Debt (Absolute Difference in Sleep Duration Between Work and Free Days) Among US Adults Aged 20 Years or Older, NHANES 2017-2020[a]

Characteristics	Sleep debt Mean (95% CI)	*P* value[b]	0-1h, % (95% CI)	*P* value[b]	1-2h % (95% CI)	*P* value[b]	≥ 2h % (95% CI)	*P* value[b]
**Overall.[c]**	1.02 (0.96 - 1.08)		50.8 (48.6 - 53.0)		26.5 (25.2 - 27.8)		22.7 (20.7 - 24.8)	
**Age (years)**								
20-39	1.29 (1.22 - 1.36)	<.001	38.6 (35.9 - 41.4)	<.001	31.6 (29.1 - 34.0)	<.001	29.8 (27.6 - 32.1)	<.001
40-59	1.16 (1.58 - 1.25)		44.4 (41.2 - 47.6)		28.9 (26.1 - 31.6)		26.7 (23.4 - 30.1)	
60-64	0.77 (0.66 - 0.87)		60.1 (55.0 - 65.2)		23.3 (20.1 - 26.6)		16.6 (12.8 - 20.3)	
65-74	0.44 (0.39 - 0.50)		75.5 (72.0 - 79.1)		17.4 (13.9 - 20.9)		7.04 (5.63 - 8.46)	
≥75	0.34 (0.26 - 0.43)		82.0 (78.0 - 86.1)		12.1 (9.1 to 15.0)		5.88 (3.72 - 8.03)	
**Sex**								
Female	0.99 (0.93 - 1.15)	.09	51.4 (49.4 - 53.3)	.27	27.3 (25.5 - 29.1)	.13	21.3 (18.9 - 23.7)	.02
Male	1.04 (0.97 - 1.12)		50.1 (47.2 - 53.1)		25.6 (24.0 - 27.2)		24.2 (21.9 - 26.6)	
**Race/ethnicity**								
Hispanic	1.36 (1.28 - 1.43)	<.001	38.3 (35.7 - 40.8)	<.001	28.3 (25.5 - 31.1)		33.4 (31.0 - 35.9)	<.001
Non-Hispanic Black	1.35 (1.28 - 1.42)		38.7 (36.5 - 40.9)		28.9 (26.8 - 30.9)	.24	32.5 (30.1 - 34.9)	
Non-Hispanic White	0.87 (0.80 - 0.95)		56.2 (53.1 - 59.2)		25.6 (23.1 - 28.1)		18.3 (15.3 - 21.3)	
Other[d]	0.99 (3.91 - 1.38)		50.6 (46.8 - 54.4)		26.7 (23.5 - 29.9)		22.7 (18.9 - 26.4)	
**Educational attainment**								

© 2022 Di H et al. *JAMA Network Open.*

<High school	1.14 (1.81 - 1.29)	.003	48.9 (44.4 - 53.4)	.23	23.2 (20.9 - 25.4)	.07	27.9 (23.5 - 32.4)	<.001
High school	1.08 (0.97 - 1.20)		49.1 (44.8 - 53.4)		25.3 (22.7 - 27.9)		25.5 (21.6 - 29.5)	
>High school	0.96 (0.92 - 1.91)		51.8 (49.4 - 54.2)		27.6 (25.5 - 29.7)		20.6 (18.7 - 22.5)	
**Family income level (IPR)**								
<1.30	1.07 (1.98 - 1.16)	.04	51.8 (48.1 - 55.5)	.61	22.4 (20.0 - 24.8)	.02	25.7 (23.4 - 28.0)	.005
1.30-3.49	1.08 (1.98 - 1.18)		49.6 (45.7 - 53.5)		25.8 (23.5 - 28.0)		24.6 (21.2 - 28.0)	
≥3.50	0.95(087 - 1.12)		51.7 (48.5 - 54.9)		28.5 (25.8 - 31.2)		19.8 (16.7 - 22.9)	
**Work status**								
Non-employed	0.60 (0.55 - 0.66)	<.001	67.6 (64.8 - 70.5)	<.001	19.8 (17.8 - 21.7)	<.001	12.6 (11.0 - 14.2)	<.001
Part-time (1–34 hours/week)	0.96 (0.86 - 1.06)		52.6 (48.5 - 56.7)		27.7 (24.5 - 31.0)		19.7 (16.8 - 22.5)	
Full-time (≥ 35 hours/week)	1.36 (1.28 - 1.44)		36.9 (33.8 - 40.1)		31.3 (28.5 - 34.1)		31.7 (28.6 - 34.9)	
**Work schedule**								
Traditional 9 AM to 5 PM day	1.09 (1.01 - 1.18)	<.001	43.5 (40.3 - 46.8)	<.001	33.9 (31.1 - 36.7)	<.001	22.6 (19.2 - 25.9)	<.001
Regular shift work	1.72 (1.58 - 1.86)		29.5 (25.1 - 33.9)		27.5 (23.9 - 31.0)		43.1 (37.9 - 48.2)	
Rotating shift work	1.14 (1.05 - 1.23)		45.2 (41.7 - 48.7)		28.5 (25.3 - 31.7)		26.3 (22.6 - 30.0)	
Did not work	0.59 (0.54 - 0.64)		68.6 (65.9 - 71.3)		19.2 (17.4 - 21.0)		12.2 (10.5 - 13.8)	

[a] Weighted estimates and 95% CIs were estimated for each stratums. All estimates were weighted to be nationally representative.

[b] P value for overall differences across stratums.

© 2022 Di H et al. *JAMA Network Open.*

[c] Sleep debt was calculated as the absolute difference between sleep duration (duration from sleep onset time until wake time) on free days minus the weekly average sleep duration (approximated by averaging 5 work and 2 free days).

[d] “Other” includes race/ethnicity other than non-Hispanic White, non-Hispanic Black, or Hispanic.

© 2022 Di H et al. *JAMA Network Open.*

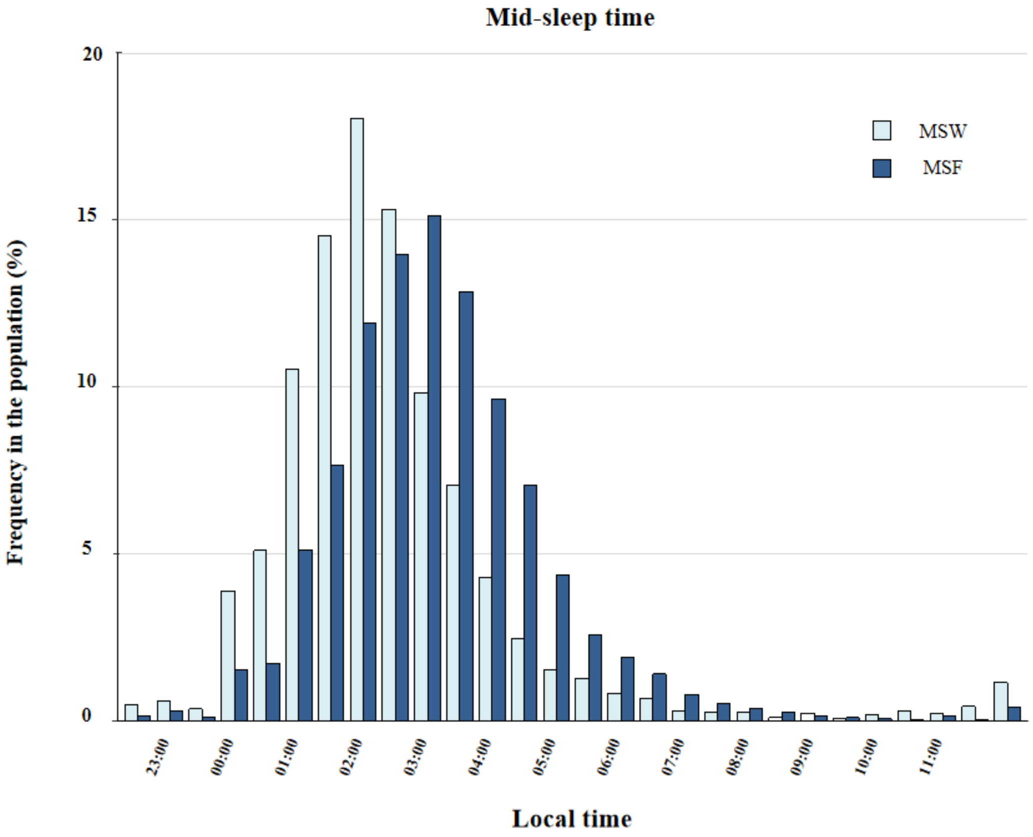

eFigure 2. Distributions of Mid-Sleep Time: Mid-Sleep on Work Days (MSW) and Free Days (MSF) With No Adjustments

The distribution is based on half-hourly bins, except for the categories at both ends. The lowest and highest bins of mid-sleep represent ≤ 23:00 and > 12:00, respectively; intermediary bins exclude the lower and include the upper limit.

© 2022 Di H et al. *JAMA Network Open.*

eTable 8. Estimated Means and Distributions of Social Jetlag (Sleep Midpoint Difference on Free-Days vs Work-Days) Among US Adults Aged 20 Years or Older, NHANES 2017-2020[a]

Characteristics	SJL, Mean (95% CI)	*P* value[b]	0-1 h, % (95% CI)	*P* value[b]	1-2 h, % (95% CI)	*P* value[b]	≥2 h, % (95% CI)	*P* value[b]
**Overall.[c]**	1.10 (1.05 - 1.15)		53.5 (50.6 - 56.4)		27.2 (24.9 - 29.5)		19.3 (17.7 - 20.9)	
**Age (years)**								
20-39	1.55 (1.44 - 1.65)	<.001	34.8 (31.6 - 38.0)	<.001	34.9 (31.8 - 38.0)	<.001	30.4 (27.5 - 33.2)	<.001
40-59	1.20 (1.11 - 1.28)		47.2 (43.2 - 51.2)		33.2 (29.7 - 36.7)		19.6 (17.0 - 22.1)	
60-74	0.53 (0.44 - 0.62)		80.2 (77.4 - 83.1)		12.9 (10.4 - 15.3)		6.90 (4.97 - 8.84)	
≥75	0.21 (0.16 - 0.26)		91.7 (89.6 - 93.8)		6.52 (4.65 - 8.40)		1.79 (0.85 - 2.72)	
**Sex**								
Female	1.04 (0.97 - 1.11)	.02	55.9 (52.6 - 59.2)	.008	26.9 (24.5 - 29.3)	.70	17.2 (15.2 - 19.2)	<.001
Male	1.16 (1.59 - 1.24)		51.0 (47.4 - 54.6)		27.5 (24.5 - 30.5)		21.5 (19.7 - 23.3)	
**Race/ethnicity**								
Hispanic	1.34 (1.22 - 1.46)	<.001	42.9 (38.9 - 47.0)	<.001	30.3 (27.5 - 33.1)	.04	26.8 (23.4 - 30.2)	<.001
Non-Hispanic Black	1.49 (1.46 - 1.59)		43.6 (41.1 - 46.1)		26.4 (24.5 - 28.3)		30.0 (27.6 - 32.4)	
Non-Hispanic White	0.98 (0.89 - 1.46)		58.1 (54.2 - 62.0)		26.3 (23.3 - 29.3)		15.6 (13.4 - 17.8)	
Hispanic								
Other[d]	1.03 (0.94 - 1.13)		52.8 (49.2 - 56.3)		28.9 (25.2 - 32.7)		18.3 (14.6 - 22.0)	
**Educational attainment**								
<High school	1.03 (0.92 - 1.16)		55.7 (51.8 - 59.7)		22.5 (19.9 - 25.1)		21.8 (18.1 - 25.4)	

© 2022 Di H et al. *JAMA Network Open.*

High school	1.12 (1.16 - 1.19)	.08	54.5 (50.7 - 58.2)	.40	23.7 (21.0 - 26.3)	<.001	21.8 (19.4 - 24.3)	<.001
>High school	1.10 (1.03 - 1.17)		52.7 (49.0 - 56.4)		29.6 (26.6 - 32.5)		17.7 (16.0 - 19.4)	
**Family income level (IPR)**								
<1.30	1.10 (1.73 - 1.18)		53.3 (50.5 - 56.2)		25.8 (23.3 - 28.4)		20.8 (18.5 - 23.1)	
1.30-3.49	1.13 (1.05 - 1.21)	.16	54.9 (51.1 - 58.7)	.78	24.7 (22.2 - 27.2)	<.001	20.4 (17.9 - 22.9)	.006
≥3.50	1.05 (0.96 - 1.15)		52.7 (47.9 - 57.5)		30.6 (26.8 - 34.3)		16.7 (14.0 - 19.4)	
**Work status**								
Non-employed	0.59 (0.53 - 0.65)	<.001	73.9 (71.3 - 76.6)	<.001	17.0 (15.4 - 18.6)	<.001	9.05 (7.16 - 10.9)	<.001
Part-time (1–34 hours/week)	1.08 (0.95 - 1.22)		53.2 (49.0 - 57.3)		30.1 (26.3 - 33.9)		16.7 (13.3 - 20.1)	
Full-time (≥ 35 hours/week)	1.50 (1.42 - 1.59)		37.6 (34.4 - 40.7)		34.3 (31.3 - 37.3)		28.1 (26.0 - 30.3)	
**Work schedule**								
Traditional 9 AM to 5 PM day	1.19 (1.11 - 1.27)	<.001	41.0 (36.9 - 45.1)	<.001	37.8 (33.6 - 42.0)	<.001	21.2 (18.0 - 24.4)	<.001
Regular shift work	2.21 (1.92 - 2.49)		33.1 (28.4 - 37.8)		25.9 (22.6 - 29.2)		41.0 (36.6 - 45.5)	
Rotating shift work	1.13 (1.04 - 1.21)		47.0 (42.2 - 51.9)		33.2 (29.1 - 37.3)		19.8 (16.7 - 22.8)	
Did not work	0.56 (0.50 - 0.62)		75.5 (72.9 - 78.2)		15.9 (14.5 - 17.3)		8.57 (6.68 - 10.5)	

[a] Weighted estimates and 95% CIs were estimated for each stratums. All estimates were weighted to be nationally representative.

[b] P value for overall differences across stratums.

[c] Social jetlag (SJL) was calculated based on the absolute difference between mid-sleep time on work days and free days.

[d] “Other” includes race/ethnicity other than non-Hispanic White, non-Hispanic Black, or Hispanic.

© 2022 Di H et al. *JAMA Network Open.*

eTable 9. Weighted Logistic Regression Models of Sleep Duration Among US Adults Aged 20 Years or Older, Adjusted for Sociodemographic and Employment Characteristics, NHANES 2017-2020[a]

Characteristics	Adjusted odds ratio (95% CI)[b]			
	Work days		Free days	
	Sleeping <7 h	Sleeping ≥9 h	Sleeping <7 h	Sleeping ≥9 h
**No.[c]**	2260	2062	1376	3637
**Age (years)**				
20-39	1 [Reference]	1 [Reference]	1 [Reference]	1 [Reference]
40-59	1.37 (1.20 - 1.57)	0.62 (0.50 - 0.75)	1.49 (1.21 - 1.84)	0.74 (0.64 - 0.85)
60-74	1.21 (0.96 - 1.51)	0.70 (0.54 - 0.92)	1.70 (1.28 - 2.24)	0.51 (0.41 - 0.63)
≥75	0.83 (0.61 - 1.14)	0.98 (0.72 - 1.35)	1.17 (0.88 - 1.57)	0.65 (0.50 - 0.84)
P for trend[d]	.13	.08	<.001	<.001
**Sex**				
Female	1 [Reference]	1 [Reference]	1 [Reference]	1 [Reference]
Male	1.39 (1.22 - 1.59)	0.74 (0.63 - 0.87)	1.54 (1.31 - 1.80)	0.66 (0.60 - 0.73)
**Race/ethnicity**				
Hispanic	1.16 (0.93 - 1.44)	1.05 (0.87 - 1.26)	0.94 (0.71 - 1.26)	1.44 (1.20 - 1.73)
Non-Hispanic Black	1.67 (1.39 - 2.01)	1.15 (0.99 - 1.35)	1.90 (1.57 - 2.29)	1.02 (0.92 - 1.14)
Non-Hispanic White	1 [Reference]	1 [Reference]	1 [Reference]	1 [Reference]
Other[f]	1.17 (0.95 - 1.43)	1.08 (0.89 - 1.30)	1.12 (0.83 - 1.51)	1.12 (0.98 - 1.27)

© 2022 Di H et al. *JAMA Network Open.*

**Educational attainment**				
<High school	1 [Reference]	1 [Reference]	1 [Reference]	1 [Reference]
High school	0.98 (0.76 - 1.27)	0.96 (0.72 - 1.28)	1.16 (0.84 - 1.58)	0.89 (0.74 - 1.08)
>High school	0.98 (0.75 - 1.29)	0.81 (0.64 - 1.02)	0.97 (0.75 - 1.26)	0.72 (0.61 - 0.86)
P for trend[e]	.91	.02	.31	<.001
**Family income level (IPR)**				
<1.30	1 [Reference]	1 [Reference]	1 [Reference]	1 [Reference]
1.30-3.49	0.92 (0.74 - 1.16)	0.83 (0.69 - 0.99)	0.80 (0.66 - 0.97)	1.05 (0.91 - 1.20)
≥3.50	0.82 (0.62 - 1.08)	0.62 (0.48 - 0.80)	0.51 (0.39 - 0.66)	0.91 (0.76 - 1.08)
P for trend[d]	0.15	<.001	<.001	.08
**Work status**				
Non-employed	1.36 (0.73 - 2.52)	0.71 (0.41 - 1.24)	1.06 (0.56 - 2.03)	0.77 (0.39 - 1.51)
Part-time (1–34 hours/week)	1 [Reference]	1 [Reference]	1 [Reference]	1 [Reference]
Full-time (≥ 35 hours/week)	1.67 (1.36 - 2.05)	0.47 (0.36 - 0.61)	1.21 (0.94 - 1.57)	1.10 (0.86 - 1.42)
**Work schedule**				
Traditional 9 AM to 5 PM day	1 [Reference]	1 [Reference]	1 [Reference]	1 [Reference]
Regular shift work	2.52 (1.94 - 3.27)	1.07 (0.83 - 1.39)	1.20 (0.83 - 1.74)	1.25 (1.00 - 1.57)
Rotating shift work	1.63 (1.22 - 2.18)	1.09 (0.79 - 1.50)	1.59 (1.08 - 2.33)	0.83 (0.66 - 1.04)
Did not work	1.24 (0.67 - 2.31)	2.62 (1.53 - 4.49)	1.67 (1.00 - 2.77)	1.44 (0.85 - 2.44)

[a] Weighted estimates and 95% CIs were estimated for each stratums. All estimates were weighted to be nationally representative.

© 2022 Di H et al. *JAMA Network Open.*

[b] Akaike's Information Criterion (AIC): 10624.98 (sleeping <7 h on workdays), 7845.134 (sleeping ≥9 h on workdays), 10251.69 (sleeping <7 h on free days), 12983.87 (sleeping ≥9 h on free days); Bayesian's Information Criterion (BIC): 10761.86 (sleeping <7 h on workdays), 7982.021 (sleeping ≥9 h on workdays), 10388.57 (sleeping <7 h on free days), 13120.75 (sleeping ≥9 h on free days).

[c] Unweight number of participants.

[d] Calculated using the median value of each category as a continuous variable.

[e] Calculated using education level as a continuous variable.

[f] "Other" includes race/ethnicity other than non-Hispanic White, non-Hispanic Black, or Hispanic.

© 2022 Di H et al. *JAMA Network Open.*

eTable 10. Weighted Logistic Regression Models of Sleep-Wake Timing Among US Adults Aged 20 Years or Older, Adjusted for Sociodemographic and Employment Characteristics, NHANES 2017-2020[a]

Characteristics	Adjusted odds ratio (95% CI)[b]							
	Work days				Free days			
	Before 22:00	Midnight or later	Before 6:00	8:00 or later	Before 22:00	Midnight or later	Before 6:00	8:00 or later
**No.[c]**	1799	2481	2832	2025	1150	3682	1178	4327
**Age (years)**								
20-39	1 [Reference]	1 [Reference]	1 [Reference]	1 [Reference]	1 [Reference]	1 [Reference]	1 [Reference]	1 [Reference]
40-59	1.43 (1.13 - 1.83)	0.45 (0.38 - 0.53)	1.82 (1.48 - 2.23)	0.43 (0.33 - 0.57)	2.17 (1.64 - 2.88)	0.52 (0.46 - 0.59)	3.04 (2.24 - 4.12)	0.45 (0.38 - 0.53)
60-74	1.92 (1.48 - 2.48)	0.22 (0.18 - 0.26)	2.18 (1.72 - 2.76)	0.44 (0.33 - 0.60)	3.33 (2.37 - 4.67)	0.26 (0.22 - 0.32)	6.89 (4.92 - 9.65)	0.22 (0.18 - 0.26)
75 and more	1.80 (1.44 - 2.24)	0.20 (0.17 - 0.23)	1.56 (1.11 - 2.19)	0.46 (0.36 - 0.59)	3.70 (2.78 - 4.93)	0.20 (0.16 - 0.24)	5.10 (3.44 - 7.56)	0.20 (0.17 - 0.23)
P for trend[d]	<.001	<.001	<.001	<.001	<.001	<.001	<.001	<.001
**Sex**								
Female	1 [Reference]	1 [Reference]	1 [Reference]	1 [Reference]	1 [Reference]	1 [Reference]	1 [Reference]	1 [Reference]
Male	0.80 (0.69 - 0.92)	0.92 (0.83 - 1.03)	1.03 (0.90 - 1.18)	1.05 (0.87 - 1.28)	0.83 (0.65 - 1.07)	1.53 (1.31 - 1.78)	1.21 (1.00 - 1.48)	0.92 (0.83 - 1.03)
**Race/ethnicity**								

© 2022 Di H et al. *JAMA Network Open.*

Non-Hispanic Black	1.11 (0.93 - 1.31)	1.24 (1.07 - 1.44)	1.12 (0.96 - 1.31)	0.91 (0.76 - 1.08)	1.19 (0.95 - 1.48)	1.47 (1.24 - 1.74)	1.14 (0.91 - 1.43)	1.24 (1.07 - 1.44)
Non-Hispanic White	1 [Reference]	1 [Reference]	1 [Reference]	1 [Reference]	1 [Reference]	1 [Reference]	1 [Reference]	1 [Reference]
Other[f]	0.62 (0.46 - 0.85)	1.46 (1.18 - 1.81)	0.75 (0.62 - 0.92)	1.11 (0.87 - 1.41)	0.70 (0.48 - 1.02)	1.36 (1.06 - 1.74)	0.79 (0.59 - 1.08)	1.46 (1.18 - 1.81)
**Educational attainment**								
<High school	1 [Reference]	1 [Reference]	1 [Reference]	1 [Reference]	1 [Reference]	1 [Reference]	1 [Reference]	1 [Reference]
High school	0.97 (0.74 - 1.26)	0.97 (0.79 - 1.19)	0.91 (0.70 - 1.20)	1.12 (0.94 - 1.33)	0.82 (0.61 - 1.09)	1.12 (0.92 - 1.37)	1.10 (0.80 - 1.53)	0.97 (0.79 - 1.19)
>High school	0.66 (0.54 - 0.81)	1.00 (0.84 - 1.19)	0.63 (0.49 - 0.83)	1.07 (0.85 - 1.35)	0.55 (0.43 - 0.72)	1.26 (1.05 - 1.51)	0.76 (0.58 - 1.00)	1.00 (0.84 - 1.19)
P for trend[e]	<.001	.12	<.001	.005	<.001	.26	.006	.51
**Family income level (IPR)**								
<1.30	1 [Reference]	1 [Reference]	1 [Reference]	1 [Reference]	1 [Reference]	1 [Reference]	1 [Reference]	1 [Reference]
1.30-3.49	0.77 (0.64 - 0.94)	1.22 (1.01 - 1.49)	0.96 (0.79 - 1.16)	1.03 (0.82 - 1.30)	0.92 (0.72 - 1.17)	0.97 (0.79 - 1.20)	0.71 (0.55 - 0.91)	1.22 (1.01 - 1.49)
≥3.50	0.75 (0.58 - 0.97)	0.93 (0.73 - 1.19)	1.02 (0.83 - 1.25)	0.68 (0.51 - 0.91)	0.86 (0.67 - 1.11)	0.75 (0.59 - 0.94)	0.71 (0.57 - 0.89)	0.93 (0.73 - 1.19)
P for trend[d]	.13	<.001	.26	.004	.55	.003	.09	.06
**Work status**								
Non-employed	1.06 (0.53 - 2.11)	1.12 (0.79 - 1.57)	1.96 (1.06 - 3.62)	0.74 (0.39 - 1.41)	0.74 (0.29 - 1.89)	1.07 (0.78 - 1.48)	0.81 (0.32 - 2.05)	1.12 (0.79 - 1.57)

© 2022 Di H et al. *JAMA Network Open.*

Part-time (1–34 hours/week)	1 [Reference]	1 [Reference]	1 [Reference]	1 [Reference]	1 [Reference]	1 [Reference]	1 [Reference]	1 [Reference]
Full-time (≥ 35 hours/week)	1.66 (1.23 - 2.24)	0.91 (0.71 - 1.15)	2.74 (2.01 - 3.75)	0.54 (0.43 - 0.67)	1.33 (0.95 - 1.88)	0.79 (0.62 - 1.02)	1.31 (0.88 - 1.96)	0.91 (0.71 - 1.15)
**Work schedule**								
Traditional 9 AM to 5 PM day	1 [Reference]	1 [Reference]	1 [Reference]	1 [Reference]	1 [Reference]	1 [Reference]	1 [Reference]	1 [Reference]
Evening or nights	0.46 (0.31 - 0.69)	2.30 (1.70 - 3.11)	0.11 (0.07 - 0.16)	64.1 (40.4 - 101.8)	1.47 (0.85 - 2.53)	2.10 (1.55 - 2.85)	0.49 (0.29 - 0.84)	2.30 (1.70 - 3.11)
Early morning	2.50 (1.81 - 3.47)	0.74 (0.59 - 0.93)	8.29 (5.61 - 12.2)	0.78 (0.40 - 1.49)	2.12 (1.42 - 3.18)	0.65 (0.46 - 0.91)	1.80 (1.24 - 2.62)	0.74 (0.59 - 0.93)
Rotating shift work	0.78 (0.61 - 1.01)	1.08 (0.87 - 1.35)	1.08 (0.85 - 1.37)	5.01 (3.81 - 6.59)	1.08 (0.79 - 1.47)	1.26 (1.09 - 1.47)	1.24 (0.87 - 1.75)	1.08 (0.87 - 1.35)
Did not work	0.99 (0.48 - 2.05)	1.09 (0.77 - 1.54)	0.51 (0.26 - 1.00)	9.08 (4.34 - 19.0)	1.85 (0.67 - 5.10)	1.10 (0.79 - 1.53)	1.55 (0.56 - 4.25)	1.09 (0.77 - 1.54)

[a] Weighted estimates and 95% CIs were estimated for each stratums. All estimates were weighted to be nationally representative.

[b] Akaike's Information Criterion (AIC): 9471.818 (slept before 22:00 on workdays), 11337.17 (slept at midnight or later on workdays), 11282.23 (woke up before 06:00 on workdays), 10011.9 (woke up at 08:00 or later on wrokdays), 6850.334 (slept before 22:00 on free days), 12661.57 (slept at midnight or later on free days), 6689.295 (woke up before 06:00 on free days), 12385.43 (woke up at 08:00 or later on free days); Bayesian's Information Criterion (BIC): 9608.705 (slept before 22:00 on workdays), 11474.06 (slept at midnight or later on workdays), 11419.12 (woke up before 06:00 on workdays), 10148.79 (woke up at 08:00 or later on wrokdays), 6987.222 (slept before 22:00 on free days), 12798.45 (slept at midnight or later on free days), 6826.183 (woke up before 06:00 on free days), 12522.32 (woke up at 08:00 or later on free days).

[c] Unweight number of participants.

[d] Calculated using the median value of each category as a continuous variable.

© 2022 Di H et al. *JAMA Network Open.*

[e] Calculated using education level as a continuous variable.

[f] “Other” includes race/ethnicity other than non-Hispanic White, non-Hispanic Black, or Hispanic.

© 2022 Di H et al. *JAMA Network Open.*

eTable 11. Weighted Logistic Regression Models of Sleep Debt and Social Jetlag Among US Adults Aged 20 Years or Older Adjusted for Sociodemographic and Employment Characteristics, NHANES 2017-2020[a]

Characteristics	Adjusted odds ratio (95% CI)[b]			
	Sleep debt		Social jetlag	
	0 h	≥2 h	0 h	≥2 h
**No.**[c]	3535	2203	2602	1634
**Age (years)**				
20-39	1 [Reference]	1 [Reference]	1 [Reference]	1 [Reference]
40-59	1.31 (1.20 - 1.43)	1.04 (0.78 - 1.38)	1.74 (1.44 - 2.09)	0.56 (0.45 - 0.69)
60-74	2.64 (2.23 - 3.13)	0.44 (0.33 - 0.58)	4.59 (3.74 - 5.62)	0.24 (0.17 - 0.34)
≥75	4.06 (3.08 - 5.35)	0.32 (0.17 - 0.61)	6.91 (5.16 - 9.27)	0.08 (0.04 - 0.14)
P for trend[d]	<.001	<.001	<.001	<.001
**Sex**				
Female	1 [Reference]	1 [Reference]	1 [Reference]	1 [Reference]
Male	1.25 (1.14 - 1.37)	0.98 (0.78 - 1.24)	1.24 (1.07 - 1.43)	1.08 (0.94 - 1.24)
**Race/ethnicity**				
Hispanic	0.71 (0.59 - 0.84)	1.51 (1.14 - 2.01)	0.72 (0.59 - 0.87)	1.33 (1.04 - 1.71)
Non-Hispanic Black	0.62 (0.51 - 0.74)	1.82 (1.42 - 2.33)	0.62 (0.52 - 0.73)	1.93 (1.60 - 2.34)
Non-Hispanic White	1 [Reference]	1 [Reference]	1 [Reference]	1 [Reference]

© 2022 Di H et al. *JAMA Network Open.*

Other[f]	0.93 (0.79 - 1.11)	1.07 (0.75 - 1.51)	0.99 (0.81 - 1.20)	1.04 (0.74 - 1.46)
**Educational attainment**				
<High school	1 [Reference]	1 [Reference]	1 [Reference]	1 [Reference]
High school	1.02 (0.81 - 1.30)	0.85 (0.61 - 1.18)	1.07 (0.86 - 1.33)	0.97 (0.81 - 1.16)
>High school	1.08 (0.88 - 1.33)	0.61 (0.42 - 0.87)	1.08 (0.89 - 1.33)	0.73 (0.57 - 0.94)
P for trend[e]	.42	.007	.55	.006
**Family income level (IPR)**				
<1.30	1 [Reference]	1 [Reference]	1 [Reference]	1 [Reference]
1.30-3.49	0.82 (0.66 - 1.00)	1.08 (0.74 - 1.56)	0.82 (0.66 - 1.02)	1.11 (0.87 - 1.40)
≥3.50	0.75 (0.63 - 0.90)	0.65 (0.44 - 0.95)	0.68 (0.55 - 0.83)	0.97 (0.74 - 1.27)
P for trend[d]	.02	.004	<.001	.38
**Work status**				
Non-employed	1.20 (0.57 - 2.52)	0.93 (0.37 - 2.35)	0.83 (0.46 - 1.71)	1.41 (0.83 - 2.40)
Part-time (1–34 hours/week)	1 [Reference]	1 [Reference]	1 [Reference]	1 [Reference]
Full-time (≥ 35 hours/week)	0.54 (0.44 - 0.66)	1.82 (1.41 - 2.34)	0.53 (0.43 - 0.66)	1.93 (1.55 - 2.40)
**Work schedule**				
Traditional 9 AM to 5 PM day	1 [Reference]	1 [Reference]	1 [Reference]	1 [Reference]
Regular shift work	0.79 (0.60 - 1.04)	2.84 (2.02 - 4.00)	0.96 (0.72 - 1.29)	2.37 (1.71 - 3.29)
Rotating shift work	1.21 (0.97 - 1.50)	1.67 (1.11 - 2.52)	1.43 (1.08 - 1.89)	0.98 (0.74 - 1.31)
Did not work	1.51 (0.71 - 3.21)	1.08 (0.49 - 2.38)	2.72 (1.33 - 5.56)	0.64 (0.36 - 1.14)

© 2022 Di H et al. *JAMA Network Open.*

[a] Weighted estimates and 95% CIs were estimated for each stratums. All estimates were weighted to be nationally representative.

[b] Akaike's Information Criterion (AIC): 11510.32 (no sleep debt), 10433.72 (≥2 h of sleep debt), 10018.66 (no social jetlag), 9137.917 (≥2 h of social jetlag); Bayesian's Information Criterion (BIC): 11647.21 (no sleep debt), 10570.61 (≥2 h of sleep debt), 10155.55 (no social jetlag), 9274.805 (≥2 h of social jetlag).

[c] Unweight number of participants.

[d] Calculated using the median value of each category as a continuous variable.

[e] Calculated using education level as a continuous variable.

[f] "Other" includes race/ethnicity other than non-Hispanic White, non-Hispanic Black, or Hispanic.

© 2022 Di H et al. *JAMA Network Open.*

eReferences

1. Mokhlesi B, Temple KA, Tjaden AH, et al. Association of Self-Reported Sleep and Circadian Measures With Glycemia in Adults With Prediabetes or Recently Diagnosed Untreated Type 2 Diabetes. *Diabetes Care.* 2019;42(7):1326-1332.
2. Roenneberg T, Allebrandt KV, Merrow M, Vetter C. Social jetlag and obesity. *Current biology : CB.* 2012;22(10):939-943.
3. Roenneberg T, Pilz LK, Zerbini G, Winnebeck EC. Chronotype and Social Jetlag: A (Self-) Critical Review. *Biology.* 2019;8(3).
4. Terman JS, Terman M, Lo ES, Cooper TB. Circadian time of morning light administration and therapeutic response in winter depression. *Arch Gen Psychiatry.* 2001;58(1):69-75.

© 2022 Di H et al. *JAMA Network Open.*
